# Integrated Metabolomics and Microbial Profiling in Patients with Irritable Bowel Syndrome

**DOI:** 10.4014/jmb.2511.11041

**Published:** 2026-01-22

**Authors:** Yeongseo Kim, Mee-Hyun Lee, Seung-Ho Seo, Juhan Pak, Soobin Bae, Gayoun Lee, Gi Dae Kim, Hyun Sik Kim, Young-Ho Moon, Hong-Seok Son

**Affiliations:** 1 Department of Biotechnology, College of Life Sciences and Biotechnology, Korea University, Seoul 02841, Republic of Korea; 2 College of Korean Medicine, Dongshin University, Naju 58245, Republic of Korea; 3 Korean Medicine Convergence Research Institute, College of Korean Medicine, Dongshin University, Naju 58245, Republic of Korea; 4 Department of Food and Nutrition, Kyungnam University, Changwon 51767, Republic of Korea; 5 Department of Biology, College of Science, Kyung Hee University, Seoul 02447, Republic of Korea; 6 Mokpo Korean Medical Hospital, Dongshin University, Mokpo 58665, Republic of Korea

**Keywords:** Irritable bowel syndrome, Gut microbiota, Metabolomics, Next-generation sequencing, UPLC-QTOF-MS, GC-MS

## Abstract

This study was conducted to identify metabolic and gut microbial changes associated with the pathophysiology of irritable bowel syndrome (IBS) by comparing the plasma/urinary metabolite profiles and gut microbiota composition between healthy controls (HC) and IBS patients. There was no significant difference in overall microbial diversity; however, IBS patients showed relatively higher abundance of *Christensenellaceae* R-7 group, *Clostridium* sensu stricto 1, and *Negativibacillus*. Metabolite analysis identified statistically significant differences in 34 plasma metabolites (VIP > 1.2, *q* < 0.05). Metabolite set abundance analysis indicated that commonly disturbed metabolic pathways in both plasma and urinary metabolites were mainly related to carbohydrate, amino acid, and fatty acid metabolism. Among these metabolic pathways, fatty acid metabolism was associated with three metabolites that showed significant correlations with the discriminating gut microbial features, namely *Clostridium* sensu stricto 1, *Negativibacillus*, and *Klebsiella*. This study demonstrated that integrating the three datasets—plasma metabolites, urinary metabolites, and gut microbial communities—provides a comprehensive overview of IBS pathophysiology. Together, these findings indicate that functional interactions between discriminative gut microbial features and systemic metabolic alterations, particularly within fatty acid metabolism, may represent a mechanistic link between gut dysbiosis and the metabolic manifestations of IBS.

## Introduction

Irritable bowel syndrome (IBS) is a chronic disorder of gut-brain interaction (DGBI) affecting approximately 4–10% of the global population [1]. IBS is considered a collection of symptoms that exhibit inter-individual heterogeneity and intra-individual temporal instability, including abdominal pain, cramping, and changes in stool form or frequency [2]. The decline in quality of life experienced by IBS patients imposes a significant burden on both the patients and society. For this reason, improving the diagnosis and treatment of IBS remains an important clinical need. However, the current limited understanding of the pathophysiology of IBS poses a significant challenge to advancing research in this area [1].

Although the precise mechanism of IBS remains unclear, alterations in the gut microbiota have been proposed to mediate the pathogenesis of IBS [3]. However, the composition of the human gut microbiota is highly diverse because it is influenced by multiple factors such as pathogens, ethnicity, dietary choices, geographic location, and medication use. This heterogeneity makes it difficult to clearly define what constitutes a healthy gut microbiome versus a pathological IBS microbiome [4]. Furthermore, studies on IBS-related microbial communities often yield conflicting results, underscoring the considerable heterogeneity across investigations. Thus, rather than focusing exclusively on the gut microbiome, an integrative approach that incorporates multiple omics platforms, such as metabolomics, is essential [5].

Given this complexity, integrated bioinformatic analysis has become a powerful approach to explore the interactions between microbes and metabolites, offering new insights into their relationship and potential therapeutic targets for IBS [6]. Microbial composition can shape the colonic environment, as metabolites produced by microbes may participate in signaling, modulate the immune system, or exert antimicrobial activity. This suggests a potential role of the microbiota and microbiota-associated byproducts in IBS [3], emphasizing the importance of studies integrating gut microbial and metabolite analysis. Based on this significance, integrated analyses of gut microbiota and metabolites associated with IBS have been conducted in numerous studies up to the present. For instance, previous research has analyzed extensive serum protein panels to correlate them with fecal microbiota composition [7], or combined fecal microbiota composition assessment with metatranscriptomics and fecal metabolomics to provide additional insights into the functional characteristics of gut microbes [8]. However, most of these studies have primarily focused on a single type of biological sample, such as serum or fecal metabolites. Since the analysis of a single type of biological sample is generally insufficient for extrapolation to the whole organism, incorporating multiple biofluids or tissues is required to expand metabolome coverage and enhance the representativeness of the analysis [9]. Plasma metabolomics provides insights into the systemic characteristics of disease by offering a comprehensive overview of metabolic status through a minimally invasive and patient-friendly approach, reflecting not only gut-derived metabolites but also broader metabolic changes [10]. Urine metabolomics has recently gained attention for its ability to reflect systemic physiological and pathological states by providing comprehensive metabolic information through non-invasive methods [11, 12]. Accordingly, comprehensive investigations of human metabolite profiles, encompassing both plasma and urine, are required to clarify the link between IBS pathogenesis and gut microbiota, which have yet to be comprehensively studied.

To improve metabolome coverage, it is essential not only to incorporate multiple biofluids or tissues but also to employ multiple analytical techniques concurrently [9]. Metabolomics research must be capable of simultaneously analyzing hundreds of metabolites in complex biological samples and monitoring changes in these metabolites. However, none of the currently available analytical platforms possess the capability to fully measure the entire metabolome. This may be due to the physicochemical diversity of various metabolites, such as volatile alcohols and ketones, amino and non-amino organic acids, and hydrophobic lipids. Nevertheless, most metabolomics studies available today have been performed using a single analytical method [13]. Generally, volatile organic compounds, lipids, and derivatizable molecules are analyzed using gas chromatography-mass spectrometry (GC-MS), while most semi-polar metabolites are analyzed using liquid chromatography-mass spectrometry (LC-MS) [14]. Therefore, since the types and numbers of metabolites that can be analyzed differ between the two technologies, the combined use of GC-MS and LC-MS technologies can enhance the accurate, precise, and complete identification of metabolites [13].

Despite growing interest in the role of gut microbiota and its metabolites, our understanding of how alterations in the microbiota and associated metabolites contribute to pathophysiological mechanisms remains limited [15]. Therefore, this study aimed to conduct a comprehensive investigation by integrating metabolomic data obtained from plasma and urine using both untargeted GC-MS and LC-MS technologies. This study is expected to enhance our understanding of gut microbial and plasma/urinary metabolites alterations related to IBS and contribute to the development of diagnostic strategies integrating microbial community and metabolite information.

## Materials and Methods

### Study Cohort

This study was approved by the Institutional Review Board of Mokpo Korean Medicine Hospital, Dongshin University (DSMOH24-4) in July 2024. A total of 60 participants were enrolled in this study, comprising 30 HC and 30 IBS patients. Recruitment was conducted through online and offline recruitment advertisements. All participants provided written informed consent prior to study participation. Participants ranged in age from 22 to 64 years. IBS diagnosis was based on the Rome IV criteria. They also completed validated self-assessment questionnaires to measure the severity of their IBS symptoms as summarized in Table S1. The HC group consisted of individuals with no history of functional or organic disorders, including IBS, and who showed normal clinical health based on screening. Exclusion criteria included a history of organic bowel diseases such as Crohn's disease or Celiac disease, major abdominal surgery such as gastrectomy or cholecystectomy, or other factors deemed inappropriate by the investigator. Participant characteristics are presented in Table S2. There were no significant differences in baseline characteristics with respect to age, sex, and BMI between the two groups.

### Sample Collection

Fecal, plasma, and urine samples were collected from all participants under standardized conditions. Fecal samples were self-collected by participants at home using stool collection containers. Collected feces were stored at −80°C until analysis. Blood samples were collected via venipuncture into EDTA BD Vacutainer tubes (Becton Dickinson, USA) at the hospital. Peripheral blood mononuclear cells (PBMCs) were subsequently isolated using the SepMate PBMC Isolation Kit (STEMCELL Technologies, Canada). The isolated cells were suspended in a cell preservation solution and stored at −80°C until analysis. Urine samples were freshly collected midstream in the morning at the hospital from each participant, following an overnight fast, using sterile 50 ml conical tubes. Collected urine was stored at −80°C until analysis.

### 16S rRNA Gene Sequencing and Data Processing

Genomic DNA extraction from fecal samples was performed using the AccuFAST automated system (AccuGene Inc., Republic of Korea) according to the manufacturer's instructions. For MiSeq sequencing, bacterial genomic DNA was amplified using primers 341F and 806R, which incorporate Nextera adapter sequences targeting the V3-V4 hypervariable region of the 16S rRNA gene. Amplification of the 16S rRNA gene was performed using 25 polymerase chain reaction (PCR) cycles with KAPA HiFi HotStart ReadyMix (Roche sequencing, USA). The resulting PCR products (approximately 250 bp) were purified using HiAccuBeads (AccuGene Inc.) for next-generation sequencing (NGS) library preparation. Amplified libraries were normalized to equimolar concentrations and sequenced on an Illumina MiSeq platform using the MiSeq Reagent Kit v3 (600-cycle, Illumina, USA). All sequencing reads were corrected for amplification errors to remove noise and then used to infer accurate Amplicon Sequence Variants (ASVs) using DADA2 v1.16 [16]. Classification of ASVs was performed using a Naïve Bayes classifier trained on the SILVA rRNA reference database release. Classification at all taxonomic levels was performed using the QIIME 2 framework [17], with taxonomic names assigned by referencing the SILVA database version 138.1 [18]. For *α*- and *β*-diversity analyses, sequencing depth was rarefied, and taxonomic relative abundance tables were generated from normalized counts.

### Statistical Analyses and Identification of Discriminating Gut Microbial Features

Statistical analyses and data visualization were performed using R version 4.3.3. Diversity indices were calculated using the ape [19] and vegan [20] packages. Analysis of similarities (ANOSIM) was performed based on the Bray-Curtis index using the vegdist and anosim functions from the vegan [20] package. Principal coordinate analysis (PCoA) was performed based on the Bray-Curtis and Jaccard indices using the vegdist and cmdscale functions from the vegan [20] package. Permutation multivariate analysis of variance (PERMANOVA) was conducted based on both the Bray-Curtis and Jaccard indices using the adonis2 function from the vegan [20] package. Taxonomic features specifically associated with the HC group and the IBS group were identified using linear discriminant analysis effect size (LEfSe) [21]. Group comparisons were performed using the Kruskal-Wallis test, and the Benjamini–Hochberg false discovery rate (FDR) was applied to adjust for multiple comparisons. Significant taxa were defined as those with *q*-values (adjusted *p*-values) < 0.05 and logarithmic linear discriminant analysis (LDA) scores > 2.5. All analysis results were visualized using ggplot2 [22] package. Subsequently, univariate *t*-tests were applied to the gut microbial features using GraphPad Prism software (version 9.4.1, GraphPad Software Inc., USA). For multiple comparison correction, *q*-values were calculated using the Benjamini–Hochberg FDR method following *t*-tests. Finally, gut microbial features identified by LEfSe [21] analysis with *p*-values < 0.05 were considered discriminating.

### UPLC-QTOF-MS Analysis and Data Processing

Non-targeted metabolite profiling of plasma samples was performed using ultra-performance liquid chromatography coupled with quadrupole time-of-flight mass spectrometry (UPLC-QTOF-MS). Instrumental conditions and metabolomic analysis workflows were adapted from a previously published metabolomics study [23] with minor modifications. The frozen plasma samples were thawed at room temperature prior to analysis. For metabolite extraction, 60 μl of plasma was mixed with 180 μl of extraction solvent (acetonitrile:methanol, 4:1, v/v). The mixture was vortexed for 5 min and subsequently subjected to ultrasonication for 20 min using a Powersonic 520 (Hwashin, Republic of Korea) to enhance metabolite release. After centrifugation at 15,947 ×*g* for 10 min at 4°C, the supernatant was collected. This centrifugation step was repeated twice to ensure complete removal of particulates. Finally, 150 μl of the clarified supernatant was transferred to a new vial for UPLC-QTOF-MS analysis.

The plasma extracts were analyzed on an ACQUITY UPLC system (Waters, USA) coupled to a Xevo G3 QTOF mass spectrometer (Waters) equipped with an electrospray ionization (ESI) source. Chromatographic separation was achieved on an ACQUITY UPLC HSS T3 column (2.1 × 100 mm, 1.8 μm). The mobile phases consisted of (A) water with 0.1% formic acid and (B) acetonitrile with 0.1% formic acid, with gradient elution as follows: 0–6 min, 5% B; 6–9 min, 5–95% B; 9–15 min, 95% B; 15–17.1 min, 95–5% B; 17.1–20 min, 5% B. The flow rate was maintained at 0.3 ml/min, and the injection volume was 2 μL.

The mass spectrometer was operated in both positive and negative ion modes with the following parameters: source temperature, 100°C; desolvation temperature, 300°C; desolvation gas flow, 600 L/h; cone gas flow, 50 L/h; capillary voltage, 2.5 kV. The scan range was set to m/z 50–1200 with a scan time of 0.2 sec. For accurate mass calibration, leucine enkephalin (m/z 556.2771 [M+H]+ and 554.2615 [M–H]−) was used as the lockmass. The low-energy function was set at 6 V, while the high-energy function was ramped from 20 to 45 V. All data were acquired in resolution mode, and dynamic range was set to normal. Quality control (QC) samples were prepared by pooling equal aliquots from each sample and analyzed at regular intervals throughout the run to monitor system stability and reproducibility.

The raw UPLC–QTOF-MS data were preprocessed using Progenesis QI (Nonlinear Dynamics, UK). To ensure analytical reliability, features were filtered through a series of quality control procedures. Features predominantly detected in blanks were removed. Precision filtering was applied using the relative standard deviation of QC samples, and features showing non-specific responses in blanks were excluded. Metabolite annotation was performed by matching accurate mass, isotope distribution, and fragmentation spectra against established public databases. Only candidates with annotation scores above the defined threshold were retained for further evaluation. Final identifications were accepted when key criteria including mass accuracy, fragmentation pattern agreement, and isotope similarity were satisfied. Subsequently, these metabolites were structurally identified and interpreted through exact mass searches in metabolite-related databases such as HMDB (https://www.hmdb.ca/, Accessed Oct. 28, 2025) and PubChem (https://pubchem.ncbi.nlm.nih.gov/, Accessed Oct. 28, 2025).

### GC-MS Analysis and Data Processing

Untargeted metabolite profiling of plasma and urine samples was performed using GC-MS. The GC-MS conditions and metabolomic analysis protocol were adapted with minor modifications from previously published metabolomics studies [24, 25]. The frozen plasma samples were thawed at room temperature prior to analysis. To 40 μl of plasma, 250 μl of 70% methanol was added, and the mixture was vortexed for 5 min, followed by incubation at 37°C with shaking at 0.53 ×*g* for 30 min. The supernatant (210 μl) obtained after centrifugation at 4°C at 13,579 ×*g* for 5 min was then mixed with 20 μl of ribitol solution (internal standard, 0.5 mg/ml, distilled water-based). The mixture was dried under reduced pressure using the same vacuum drying chamber (HyperVAC, Republic of Kore).

The frozen urine samples were thawed at room temperature prior to analysis. To 100 μl of urine, 900 μl of methanol was added, and the mixture was thoroughly vortexed for 5 min. The supernatant (400 μl) obtained after centrifugation at 15,947 ×*g* for 10 min at 4°C was mixed with 20 μl of ribitol solution (internal standard, 0.5 mg/ml, distilled water-based). The mixture was dried under reduced pressure using the same vacuum drying chamber.

To evaluate the stability and reliability of the analysis, quality control (QC) samples were prepared by mixing 30 μl of each sample. After vacuum drying, samples were treated with 100 μl of O-methoxyamine hydrochloride solution (20 mg/ml, pyridine-based) and sonicated for 20 min at 4°C using a Powersonic 520 (Hwashin). The mixture was then vortexed for 1.5 min and incubated for 90 min at 30°C in the dark at 0.53 ×*g*. Subsequently, the sample was silylated with 50 μl of N-methyl-N-(trimethylsilyl) trifluoroacetamide (MSTFA) and reacted for 30 min at 37°C at 0.53 ×*g*. After the reaction, the mixture was centrifuged at 4°C at 13,579 ×*g* for 5 min. The supernatant (80 μl) was transferred to a 2 ml glass vial equipped with a 100 μl insert for metabolite analysis.

Derivatized samples were analyzed using a GC–MS (QP2020, Shimadzu, Japan) equipped with an RTX-5MS capillary column (Restek, USA). The GC–MS conditions were as follows: Injector temperature 230°C, transfer line temperature 250°C, helium flow rate 1 ml/min, detector temperature 280°C. The GC oven temperature program started at 80°C, held for 2 min, then ramped at 15°C/min to reach the final temperature of 330°C, which was held for 6 min. To ensure analytical stability, performance, and reproducibility, QC samples were analyzed alongside the test samples.

The retention index (RI) values of the compounds were calculated using the retention times (RT) of C7–C40 alkane standards (Sigma-Aldrich, USA) under the same GC–MS conditions. Raw data were processed using GCMS solution Postrun Analysis (Shimadzu) and MS-DIAL v4.9, and converted to ABF format. Compound identification was performed by referencing electron impact (EI) spectra and Kovats RI, applying a retention index tolerance of 20, an EI similarity criterion of 90%, and an identification score criterion of 90%. Identified spectra were additionally compared against the NIST v. 20.0 library and standard reagents. Peak intensities were normalized based on total ion count. Peak intensities were normalized to ribitol, and subsequently normalized by total peak area (TPA, sum normalization) to account for differences in overall concentration of plasma and urine. To improve data quality, features with an average intensity <1 in QC or blank samples were excluded, and metabolites with poor reproducibility (QC %RSD >30%) across pooled QC samples were excluded prior to statistical analysis.

### Statistical Analysis and Identification of Discriminating Metabolites

The processed datasets were subjected to multivariate statistical analysis, with partial least squares discriminant analysis (PLS-DA) performed using SIMCA v.18.0 (Umetrics, Sweden). The PLS-DA model was considered valid if both the *R*^2^Y and *Q*^2^Y intercepts from the permutation test did not exceed 0.3–0.4 and 0.05, respectively [26]. Subsequently, to identify significant metabolites contributing to classification, metabolites with a variable importance in projection (VIP) values > 1.2 were selected from the PLS-DA loading plot. Univariate analysis using the *t*-test was then applied to these metabolites. The *t*-tests were performed using GraphPad Prism software (version 9.4.1, GraphPad Software Inc., USA). For multiple comparison correction, *q*-values were calculated using the Benjamini–Hochberg FDR method following *t*-tests. Finally, metabolites with a VIP score > 1.2 and a *q*-values < 0.05 were considered discriminating metabolites.

### Metabolite Set Enrichment Analysis (MSEA) Using MetaboAnalyst Software

Analysis of metabolic pathways associated with all metabolites was performed using quantitative enrichment analysis in MetaboAnalyst 6.0 (https://www.metaboanalyst.ca/, Accessed Oct. 30, 2025). For plasma, processed GC-MS data and LC-MS data from both positive and negative modes were standardized using *Z*-scores and then combined for use. For urine, processed GC-MS data were used.

### Correlation Analysis between Discriminating Metabolic and Gut Microbial Features

Discriminating plasma/urinary metabolic and gut microbial features were visualized as heatmaps using Spearman's correlation coefficient. At the genus level, the correlation strength between each identified gut microbiota and the detected metabolites was color-mapped using the Spearman's correlation coefficient(*ρ*). R values were represented by various colors, with the color legend indicating different significance levels (*p < 0.05, **p < 0.01, ***p < 0.001).

## Results and Discussion

### Taxonomic Composition and Diversity Assessment of Gut Microbiota

**Fig. 1:**
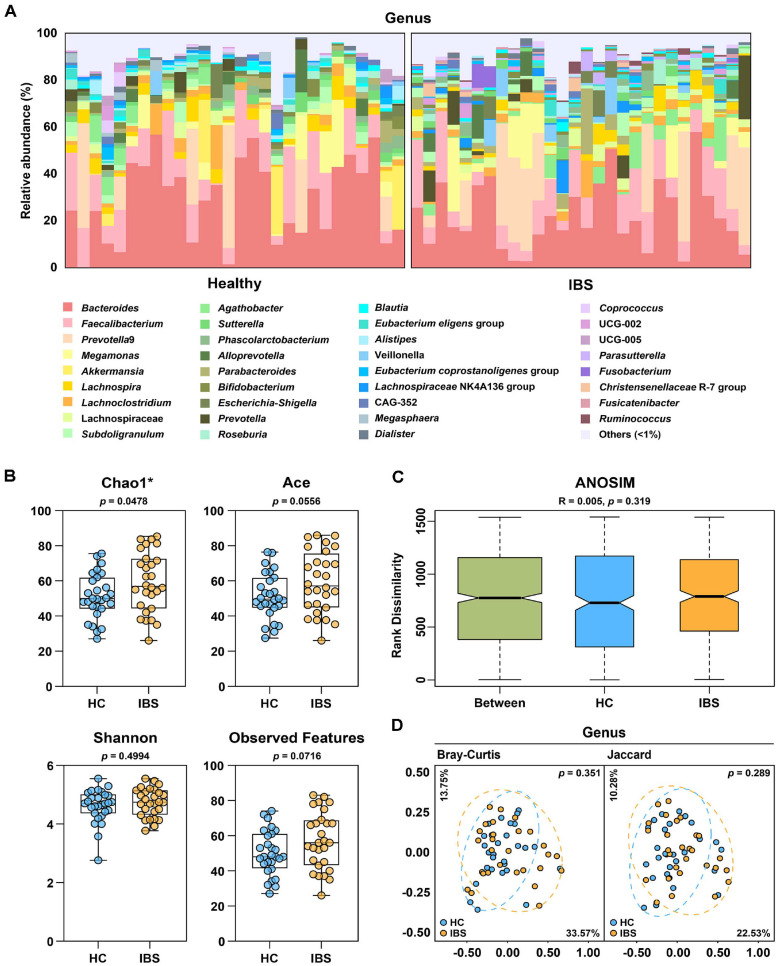
Comparative analysis of gut microbiota taxonomic structures in the HC and IBS groups. (**A**) Bar charts depicting the relative abundance of microbiota at the genus level in both the HC and IBS groups. (**B**) *α*-diversity analysis comparing the HC and IBS groups, based on Chao1, Ace, Shannon index, and Observed features. (**C**) ANOSIM similarity index of gut microbiota between the HC and IBS groups. (**D**) Principal coordinate analyses of the taxonomic structures of the gut microbiota at species and genus levels of the HC and IBS groups, based on Bray-Curtis and Jaccard indices. P-values of comparisons between the groups computed via permutational analysis of variance.

The taxonomic composition of the gut microbiota was first examined to assess differences between the HC and IBS groups. As shown in Fig. 1A, the microbial composition at the genus level exhibited inter-individual variability, but no clear distinction between the two groups was observed. The four dominant genera in both groups were identical: *Bacteroides*, *Faecalibacterium*, *Prevotella* 9, and *Megamonas*.

To further evaluate gut microbiota structure, we analyzed *α*-diversity indices including Chao1, Ace, Shannon, and observed features (Fig. 1B). The Chao1 index revealed a significant increase in species richness in the IBS group compared to the HC group (*p* < 0.05), while indices such as Ace, Shannon, and Observed Features showed no significant differences between groups. Furthermore, ANOSIM based on the Bray-Curtis index yielded an R value of 0.005 and a *p*-value of 0.326, indicating that the overall microbial community composition was similar between the two groups (Fig. 1C). *β*-diversity was assessed using Bray-Curtis and Jaccard distance matrices and visualized by PCoA to examine structural differences between groups. At genus level, substantial overlap was observed between the HC and IBS groups (Fig. 1D), with no statistically significant clustering (PERMANOVA, *p* > 0.05 for all comparisons). These results suggest that, at the microbial community level, there are no clear diversity differences between the HC and IBS groups. The corresponding raw *p*-values, *q*-values, and effect sizes (Hedges’ *g*) are provided in Table S3.

### Discriminating Taxonomic Features of Gut Microbiota

**Fig. 2:**
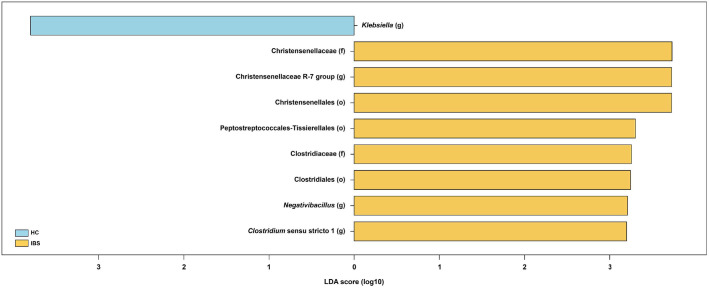
Identification of taxonomic features distinguishing the HC and IBS groups. Taxonomic features identified by LEfSe from gut microbiota16S rRNA amplicon sequencing data across all taxonomic hierarchy levels.

To identify specific bacterial taxonomic groups distinguishing the HC group from the IBS group, we performed LEfSe analysis based on 16S rRNA gene sequencing data of the gut microbiota, which included taxonomic hierarchy levels down to the genus level. Applying an LDA effect size index of 2.5 revealed that three bacterial genera (*Christensenellaceae* R-7 group, *Clostridium* sensu stricto 1, *Negativibacillus*), two bacterial families (Christensenellaceae, Clostridiaceae), and three bacterial orders (Christensenellales, Peptostreptococcales-Tissierellales, Clostridiales) were significantly enriched in the IBS group (Fig. 2). In contrast, *Klebsiella* was significantly enriched in the HC group.

### UPLC-QTOF-MS Based Plasma Metabolic Profiles and Identification of Discriminating Metabolites

**Fig. 3:**
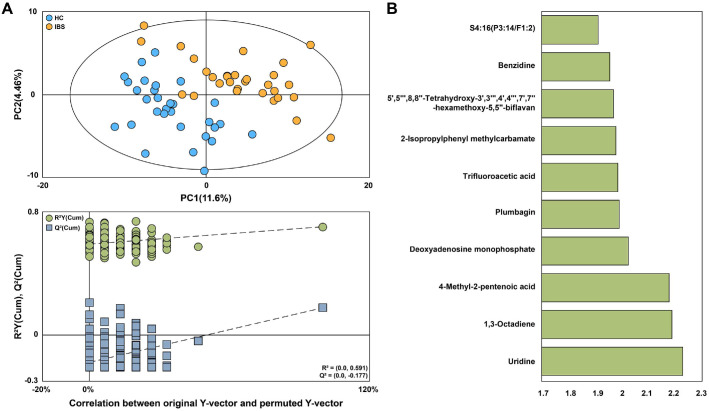
Plasma metabolites of the HC and IBS groups analyzed by UPLC-QTOF-MS. (**A**) Projection to latent structures and discriminant analysis (PLS-DA) score plot and 300 permutations test obtained from UPLC-QTOF–MS data of plasma samples of the HC and IBS groups. (**B**) Identified metabolites with VIP scores (VIP > 1.2), indicating their importance in distinguishing the groups. Figure shows only the top 10 metabolites with the highest VIP scores.

Comprehensive analysis of plasma metabolites using UPLC-QTOF-MS revealed clear separation between the HC group and the IBS group on the PLS-DA score plot, indicating distinct plasma metabolite profile differences between the two groups (Fig. 3A). In the permutation test used to evaluate the performance of the PLS-DA model, although the *R*^2^Y intercept was slightly higher than the conventional threshold of 0.4, the negative *Q*^2^Y intercept indicates that the model is not overfitted and supports the statistical validity of the group separation. A total of 110 metabolites with VIP scores exceeding 1.2 were identified as significantly contributing to group differentiation (Fig. 3B). Among these, 29 metabolites—including glucose, betaine and dodecanoic acid—showed significant differences (*q* < 0.05) between the HC and IBS groups (Table S4). The complete results of univariate testing, including raw *p*-values, *q*-values, and effect sizes (Hedges’ *g*), are provided in Tables S5 and S6.

### GC-MS Based Plasma Metabolic Profiles and Identification of Discriminating Metabolites

**Fig. 4:**
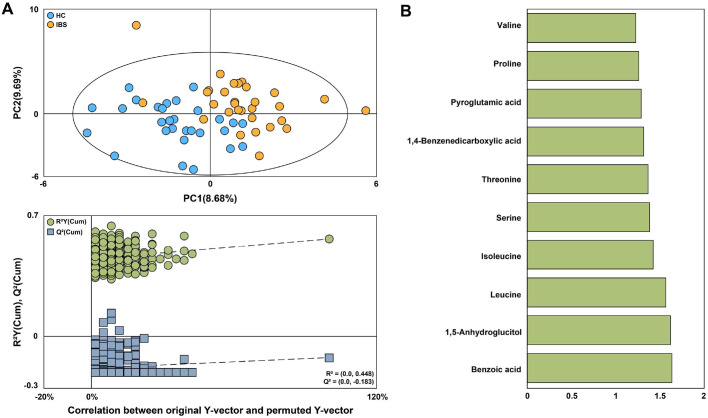
Plasma metabolites of the HC and IBS groups analyzed by GC-MS. (**A**) Projection to latent structures and discriminant analysis (PLS-DA) score plot and 300 permutations test obtained from GC–MS data of urine samples of the HC and IBS groups. (**B**) Identified metabolites with VIP scores (VIP > 1.2), indicating their importance in distinguishing the groups.

Comprehensive analysis of plasma metabolites using GC-MS revealed clear separation between the HC and IBS groups in the PLS-DA score plot. The permutation test indicated that the *R*^2^Y intercept was slightly higher than the threshold of 0.4; however, the *Q*^2^Y intercept was negative, suggesting that the PLS-DA model was not overfitted and could be considered acceptable (Fig. 4A). This indicates distinct differences in plasma metabolite profiles between the two groups. Ten metabolites—benzoic acid, 1,5-anhydroglucitol, leucine, isoleucine, serine, threonine, 1,4-benzenedicarboxylic acid, pyroglutamic acid, proline, and valine—were identified as the most significant contributors to group differences (VIP > 1.2) (Fig. 4B and Table S4). Among these, Benzoic acid and 1,5-Anhydroglucitol showed significant differences (*p* < 0.05) between the HC and IBS groups; however, neither maintained significance after FDR adjustment (q > 0.05). The complete results of univariate testing, including raw *p*-values, *q*-values, and effect sizes (Hedges’ *g*), are provided in Table S7.

### GC-MS Based Urinary Metabolic Profiles and Identification of Discriminating Metabolites

**Fig. 5:**
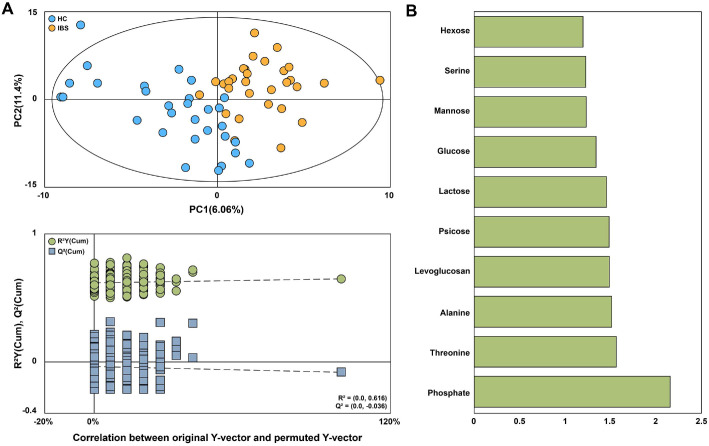
Urinary metabolites of the HC and IBS groups analyzed by GC-MS. (**A**) Projection to latent structures and discriminant analysis (PLS-DA) score plot and 300 permutations test obtained from GC–MS data of urine samples of the HC and IBS groups. (**B**) Identified metabolites with VIP scores (VIP > 1.2), indicating their importance in distinguishing the groups.

To explore metabolic differences between the HC and IBS groups, we performed a comprehensive analysis of urinary metabolites using GC-MS. Although clear separation between the HC and IBS groups was observed in the PLS-DA score plot, the permutation test indicated that the *R*^2^Y intercept exceeded 0.4. Moreover, despite the negative *Q*^2^Y intercept, the *Q*^2^ values of the permuted models on the left were higher than that of the original model on the right, indicating that the PLS-DA model was not valid (Fig. 5A). The 10 metabolites with VIP scores exceeding 1.2—phosphate, threonine, alanine, levoglucosan, psicose, lactose, glucose, mannose, serine, and hexose—were identified as significantly contributing to group separation, although none showed significant differences (*p* < 0.05) between the HC and IBS groups (Fig. 5B and Table S4). The complete results of univariate testing, including raw *p*-values, *q*-values, and effect sizes (Hedges’ *g*), are provided in Table S8.

### Key Metabolic Pathway Analysis for Plasma and Urinary Metabolites

**Fig. 6:**
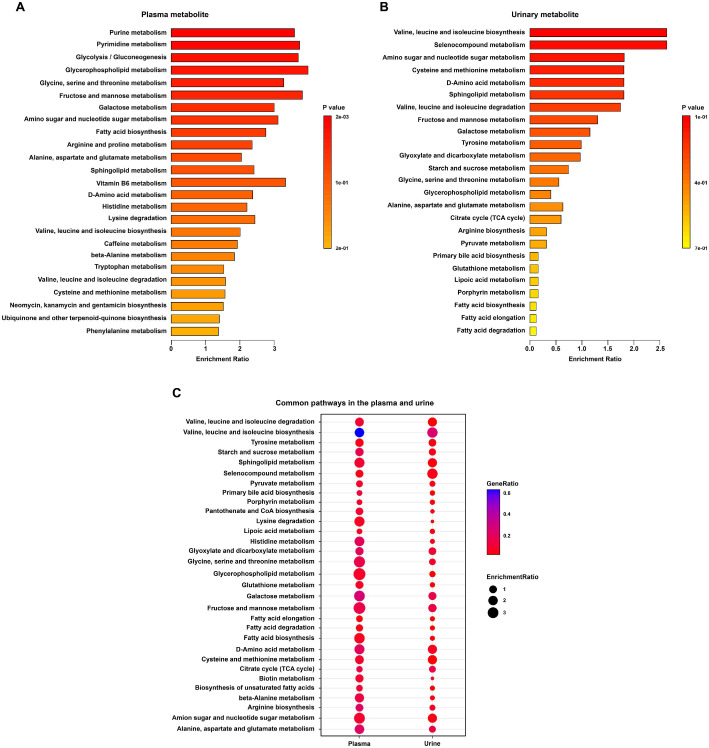
Enrichment analysis performed in MetaboAnalyst using plasma and urinary metabolite profiles from the HC and IBS groups. Overview of metabolite set enrichment for (**A**) plasma and (**B**) urine. The enrichment ratio represents the proportion of detected metabolites within each pathway. Colors indicate statistical significance, with darker red representing lower *p*-values. (**C**) KEGG pathway analysis of the dysregulated pathways in both the plasma and urine.

To elucidate metabolic changes associated with IBS, we performed MSEA on plasma and urinary metabolites. Plasma metabolite-based enrichment analysis showed several significantly enriched metabolic pathways in the IBS group (Fig. 6A). Glycerophospholipid metabolism was identified as the significantly enriched pathway with the highest enrichment ratio, consistent with previous studies that highlight the importance of glycerophospholipids in distinguishing the IBS group from the HC group [27, 28]. Enrichment analysis of urinary metabolites also showed enriched metabolic pathways in the IBS group, including valine, leucine, and isoleucine biosynthesis, which are involved in maintaining barrier integrity and protecting intestinal mucosal permeability for nutrient absorption [29] (Fig. 6B). These metabolic disturbances suggest potential mechanisms underlying IBS symptoms and warrant further investigation.

Comparison of MSEA results between plasma and urinary metabolites identified 32 commonly enriched pathways, suggesting shared metabolic alterations (Fig. 6C). Ranking these pathways by enrichment ratio revealed that many metabolic pathways associated with the identified discriminating metabolites were altered in both plasma and urine. Among the discriminating metabolites, glucose was involved in fructose and mannose metabolism, amino sugar and nucleotide sugar metabolism, galactose metabolism, and starch and sucrose metabolism. In addition, betaine and creatine were associated with glycine, serine and threonine metabolism, whereas dodecanoic acid was linked to fatty acid biosynthesis. These metabolic disturbances suggest an important role in understanding the pathogenesis of IBS symptoms.

### Correlation Analysis between Discriminating Metabolic and Gut Microbial Features

**Fig. 7:**
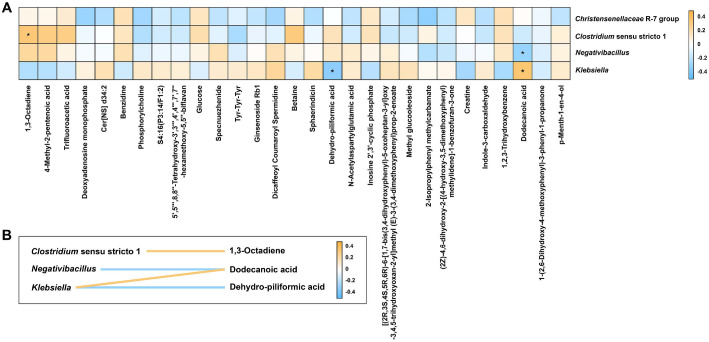
The correlation between gut microbiota composition and discriminating plasma metabolites. (**A**) The correlation heatmap identifying associations between gut microbiota composition and discriminating plasma metabolites. Orange indicates a positive correlation, while blue indicates a negative correlation. A deeper color represents a stronger correlation (*p < 0.05, **p < 0.01, ***p < 0.001). (**B**) The gut microbiota which was well predicted (*ρ* > 0.3 and < −0.3) by the metabolic variation. Associations with the metabolites are shown for each genus with the direction of correlation indicated by orange (positive) or blue (negative) lines.

The covariation between plasma discriminating metabolic and gut microbial features is presented as a heatmap (Fig. 7A). Metabolic associations (*ρ* > 0.3 and < −0.3) among well-predicted gut microbiota members were shown in Fig. 7B. Many gut microbial features at the genus level showed associations with various metabolites. The relative abundances of *Clostridium* sensu stricto 1 and *Negativibacillus* were significantly associated (*q* < 0.05) with plasma-derived 1,3-octadiene (*ρ* = 0.35) and dodecanoic acid (*ρ* = −0.30), respectively. The relative abundance of *Klebsiella* also showed significant associations (*q* < 0.05) with two plasma-derived metabolites: dehydro-piliformic acid (*ρ* = −0.33) and dodecanoic acid (*ρ* = 0.35). The three plasma-derived metabolites significantly correlated with the discriminating gut microbial features: 1,3-octadiene, dodecanoic acid and dehydro-piliformic acid are all related to fatty acids [30-32]. This suggests that the dysregulation of fatty acids in IBS patients is associated with the gut microbiota.

*Clostridium* sensu stricto 1, a representative cluster within the *Clostridium* genus, has been reported to be increased in stool samples from IBS patients [33, 34], which is consistent with the findings of the present study. This genus exhibits a consistent capacity to produce SCFAs, particularly butyrate, which promotes visceral hypersensitivity in IBS models through the mast cell-derived dorsal root ganglion (DRG) neuron lincRNA-01028-PKC-TRPV1 pathway [35]. Furthermore, SCFAs have been reported to increase lipid oxidation [36], suggesting that *Clostridium* sensu stricto 1 may have a positive correlation with 1,3-octadiene, a product of lipid oxidation. *Negativibacillus* is a normal colonizer of the human intestine. However, reports have shown that an increased abundance of this genus is associated with various pathological conditions, including the digestive tract [37]. *Negativibacillus* is known to influence the gut microecological environment, as well as intestinal barrier function and immune responses [38]. In addition, Efremova *et al*. [39] reported a positive correlation between this genus and diamine oxidase (DAO) levels. An increased concentration of circulating DAO is considered a key indicator of intestinal barrier damage resulting from enzyme leakage [40], and several previous studies have reported elevated DAO levels in IBS patients [41, 42]. Taken together, these findings and the observations of the present study suggest that the relative enrichment of *Negativibacillus* in IBS patients may be associated with intestinal barrier impairment. The strong antimicrobial activity of dodecanoic acid [43] may underlie its negative correlation with *Negativibacillus* abundance. In this study, *Klebsiella* was found to be more abundant in the HC group, which contrasts with previous reports describing its higher abundance in IBS patients [44, 45]. This discrepancy may be attributed to the substantial heterogeneity in the composition of the human gut microbiota. Meanwhile, *Klebsiella* showed a positive correlation with dodecanoic acid, which may be explained by its relative tolerance to acid mixtures, including dodecanoic acid, compared with other genera [46]. Dehydro-piliformic acid, which has a negative correlation with *Klebsiella*, is known to be a fungal metabolite [47]. Therefore, it is necessary to investigate the gut mycobiome and analyze bacterial-fungal interactions in IBS patients.

Beyond characterizing group-level differences, the integrative analysis of gut microbiota and host metabolite profiles in this study provides a conceptual framework for understanding how microbial dysbiosis may translate into systemic metabolic alterations in IBS. In particular, the convergence of discriminative microbial features and metabolites within fatty acid—related pathways suggest that microbial modulation of lipid metabolism may represent a key interface between the gut environment and host physiology. This finding extends previous microbiome-focused studies by highlighting metabolically active pathways through which gut bacteria may influence IBS pathophysiology, rather than focusing solely on taxonomic shifts. The combined profiling of gut microbial features and circulating or urinary metabolites may contribute to the development of non-invasive biomarker panels for IBS subtyping and disease monitoring. Given the heterogeneity of IBS, microbiome-metabolite signatures may enable more refined patient stratification, particularly for IBS-D and IBS-U, and could potentially inform personalized therapeutic strategies. Moreover, the metabolic pathways highlighted in this study suggest that targeted dietary modulation, postbiotic supplementation, or microbiota-directed interventions aimed at lipid and fatty acid metabolism may represent promising avenues for symptom management.

However, there are several limitations to this study. First, 16S rRNA sequencing was used, which only allowed for analysis at the genus level. To gain a more detailed understanding of the pathophysiology of IBS, species-level microbial analysis is necessary. Second, the main data and results are based on a small sample size from a single institution, which introduces the potential for bias. Therefore, further validation of the findings in cohort studies involving a larger patient population is required. Third, discriminating metabolic and gut microbial features were identified exclusively in diarrhea-predominant (IBS-D) and unclassified (IBS-U) IBS patients. Other IBS subtypes or non-IBS individuals experiencing diarrhea were not included in the analysis. Future studies comparing metabolic and microbial features across different IBS subtypes and controls could provide further insights into the pathophysiology of IBS. Finally, although we identified significant associations between gut microbiota and metabolites, these findings were based on Spearman’s rank correlation without multivariate adjustment for potential covariates such as age, sex, and BMI. Given the exploratory nature of this study and the non-normal distribution of the omics data, a non-parametric approach was prioritized to minimize Type II errors and to capture robust monotonic relationships. However, the potential influence of confounding factors cannot be entirely ruled out. Future studies with larger cohorts should employ multivariate regression models or canonical correlation analysis (CCA) to further validate these findings and to clarify the independent contributions of specific variables.

Considering these limitations, future studies that integrate shotgun metagenomics, metatranscriptomics, and mycobiome profiling in larger, multi-center cohorts will be essential to validate and extend the present findings. Such integrative multi-omics approaches will enable species- and strain-level taxonomic resolution, functional gene annotation, and a more comprehensive characterization of microbial-metabolic networks. Ultimately, these efforts may facilitate the translation of multi-omics insights into clinically actionable strategies and advance a mechanism-based understanding of IBS.

## Conclusion

This study investigated metabolic and gut microbiota changes associated with the pathophysiology of IBS by integrating plasma and urinary metabolomics with gut microbiota profiling. While no significant differences were observed in gut microbiota diversity, IBS patients showed relatively higher abundance of *Christensenellaceae* R-7 group, *Clostridium* sensu stricto 1, and *Negativibacillus*. Plasma metabolite analysis revealed distinct differences between groups in metabolites such as glucose, betaine and dodecanoic acid. However, urinary metabolite analysis did not identify significant variation of metabolites in IBS patients. MSEA indicated that among IBS-related metabolites, glycerophospholipid metabolism was the most highly enriched in plasma, whereas valine, leucine, and isoleucine biosynthesis was the most highly enriched pathway in urine. Commonly disturbed metabolic pathways in both plasma and urinary metabolites were mainly related to carbohydrate, amino acid, and fatty acid metabolism, and were also associated with discriminating metabolites. Among these metabolic pathways, fatty acid metabolism was associated with three metabolites that were significant correlated with discriminating gut microbial features, suggesting that this pathway may contribute to pathophysiology of IBS. These results highlight the importance of integrated bioinformatic analysis by combining gut microbiome and metabolite analysis in IBS research. This approach can improve our understanding of the relationship between gut microbial dysbiosis and metabolic outcomes in IBS patients, thereby providing deeper insight into the pathophysiology of IBS. Our findings suggest that the functional interplay between discriminative gut microbial features and systemic metabolic alterations—particularly within the fatty acid metabolism pathway— may serve as a potential mechanistic link between gut dysbiosis and the metabolic manifestations of IBS. However, in future research, the adoption of shotgun metagenomics and transcriptome-based analyses in larger, multi-center cohorts will be essential for elucidating species-level contributions and functional gene expression patterns.

## Supplemental Materials

Supplementary data for this paper are available on-line only at http://jmb.or.kr.
