# Supplementary material for: Integrated Metabolomics and Microbial Profiling in Patients with Irritable Bowel Syndrome
Source: J Microbiol Biotechnol. 2026 Jan 22;36:e2511041. doi: 10.4014/jmb.2511.11041 (PMC12861726; doi:10.4014/jmb.2511.11041)
Supplement: Supplementary file 1 [file jmb-36-e2511041-supple.pdf]

J. Microbiol. Biotechnol. 2026. 36: e2511041

https://doi.org/10.4014/jmb.2511.11041

Supplementary Tables

Table S1. IBS symptom assessment questionnaire used to evaluate abdominal symptoms and bowel habit disturbances.

IBS Symptom Assessment Questionnaire

Patient Name: __________ Patient ID: __________ Date: __________

Instructions: This questionnaire is designed to assess abdominal symptoms, bowel habits, and support Rome IV-based IBS subtype classification. Please answer the following questions based on your symptoms over the past 10 days. Tick the appropriate boxes or write your response where indicated.

No.	Item			Response Format
**Q1**	Have you experienced abdominal pain in the past 10 days?			□ Yes □ No
**Q2**	If yes, how severe was the abdominal pain? (0–100 scale)			Score: _______ / 100
**Q3**	On how many of the past 10 days did you experience abdominal pain?			Days: ______ / 10
**Q4**	Have you experienced abdominal bloating in the past 10 days?			□ Yes □ No
**Q5**	If yes, how severe was the bloating? (0–100 scale)			Score: _______ / 100
**Q6**	How would you describe your current bowel habits?			________________
**Q7**	Do your bowel habits interfere with your daily life?			□ Yes □ No
**Q8**	During the past 7 days, was the average intensity of your most severe abdominal pain 3.0 or higher (on a 0–10 scale)?			□ Yes □ No
**Q9**	During the past 7 days, on how many days did you have at least one stool classified as Bristol type 6 or 7?			Days: ______ / 7
**Q10**	Please record your bowel movements for the past 7 days. Include number of bowel movements and Bristol stool type(s) per day.			
	Day	**10-1** No. of Bowel Movements	**10-2** Bristol Stool Type(s)	
	1			
	2			
	3			
	4			
	5			
	6			
	7			

Table S2. Characteristics of participants in the healthy and IBS groups.

	Healthy (n=30)	IBS (n=30)	*p*-value
**Age (range)**	39.20 ± 9.19 (25-56)	38.87 ± 8.74 (22-64)	0.886
**Sex (Male / Female)**	11 (36.67%) / 19 (63.33%)	7 (23.33%) / 23 (76.67%)	0.398
**BMI**	23.61 ± 3.56	24.53 ± 4.45	0.380

Table S3. Summary of statistical testing results for gut microbial taxa at the genus level between the HC and IBS groups. The table presents raw *p*-values (Welch's *t*-test or Mann–Whitney U test), FDR-adjusted *q*-values, and effect sizes (Hedges' *g*).

No.	Genus	*p*-value	*q*-value	Hedges' *g*
1	Lachnospiraceae	0.2404	0.6608	-0.3288
2	*Eubacterium* coprostanoligenes group	0.8753	0.9882	0.0887
3	*Eubacterium* eligens group	0.3021	0.6608	0.0399
4	*Agathobacter*	0.0879	0.5128	-0.3198
5	*Akkermansia*	0.0379	0.2961	0.5208
6	*Alistipes*	0.4657	0.8150	0.2477
7	*Alloprevotella*	0.9762	0.9926	-0.0120
8	*Bacteroides*	0.1215	0.6075	0.4149
9	*Bifidobacterium*	0.7134	0.9648	0.3726
10	*Blautia*	0.4962	0.8270	0.1845
11	CAG-352	0.7957	0.9882	0.0434
12	*Christensenellaceae* R-7 group	0.0423	0.2961	-0.2091
13	*Clostridium* sensu stricto 1	0.0420	0.2961	-0.4130
14	*Coprococcus*	0.9650	0.9926	0.0831
15	*Dialister*	0.8626	0.9882	0.0589
16	*Escherichia-Shigella*	0.9623	0.9926	-0.0849
17	*Faecalibacterium*	0.6062	0.9644	0.1367
18	*Fusicatenibacter*	0.2599	0.6608	-0.3229
19	*Fusobacterium*	0.6674	0.9648	-0.2891
20	*Klebsiella*	0.0055	0.1925	0.5949
21	*Lachnoclostridium*	0.8421	0.9882	0.0795
22	*Lachnospira*	0.4012	0.7391	0.1980
23	Lachnospiraceae NK4A136 group	0.2722	0.6608	-0.3500
24	*Megamonas*	0.8753	0.9882	-0.1136
25	*Negativibacillus*	0.0275	0.2961	-0.5212
26	*Parabacteroides*	0.7167	0.9648	-0.2728
27	*Parasutterella*	0.2209	0.6608	-0.4655
28	*Phascolarctobacterium*	0.3270	0.6732	0.1255

No.	Genus	*p*-value	*q*-value	Hedges' *g*
29	*Prevotella*	0.9926	0.9926	-0.3251
30	*Prevotella* 9	0.2418	0.6608	-0.2958
31	*Roseburia*	0.7067	0.9648	-0.1592
32	*Subdoligranulum*	0.2962	0.6608	-0.1025
33	*Sutterella*	0.3570	0.6942	0.2291
34	UCG-002	0.2589	0.6608	-0.1956
35	*Veillonella*	0.1464	0.6405	-0.3282

Table S4. List of metabolites identified in plasma and urine samples with a VIP > 1.2 using UPLC-QTOF-MS and GC-MS. Metabolites with a *q* < 0.05 are highlighted.

No.	Plasma Metabolite (UPLC-QTOF-MS)	Plasma Metabolite (GC-MS)	Urine Metabolite (GC-MS)
1	Uridine	Benzoic acid	Phosphate
2	1,3-Octadiene	1,5-Anhydroglucitol	Lactose
3	4-Methyl-2-pentenoic acid	Leucine	Alanine
4	Deoxyadenosine monophosphate	Isoleucine	Levoglucosan
5	Plumbagin	Serine	Serine
6	Trifluoroacetic acid	Threonine	Threonine
7	2-Isopropylphenyl methylcarbamate	1,4-Benzenedicarboxylic acid	Glucose
8	5',5''',8,8''-Tetrahydroxy-3',3''',4',4''',7',7''-hexamethoxy-5,5''-biflavan	Pyroglutamic acid	Mannose
9	Benzidine	Proline	Psicose
10	S4:16(P3:14/F1:2)	Valine	Hexose
11	Ginsenoside Rb1		
12	Acetyl citrate		
13	Cer[NS] d34:2		
14	Dehydro-piliformic-acid		
15	Specnuezhenide		
16	Sphaerindicin		
17	Dicaffeoyl Coumaroyl Spermidine		
18	(2Z)-4,6-dihydroxy-2-[(4-hydroxy-3,5-dimethoxyphenyl)methylidene]-1-benzofuran-3-one		
19	Inosine 2',3'-cyclic phosphate		
20	[(2R,3S,4S,5R,6R)-6-[1,7-bis(4-hydroxyphenyl)-5-oxoheptan-3-yl]oxy-3,4,5-trihydroxyoxan-2-yl]methyl (E)-3-(3,4-dimethoxyphenyl)prop-2-enoate		
21	Dodecanoic acid		
22	2'-O-Methyl-5-methylcytidine		
23	Iodide		

24	N-(1-Deoxy-b-D-fructopyranosyl) (R)C(S)S-alliin
25	Betaine
26	Myristic acid
27	Serotonin
28	[(2R,3S,4S,5R,6R)-6-[1,7-bis(3,4-dihydroxyphenyl)-5-oxoheptan-3-yl]oxy-3,4,5-trihydroxyoxan-2-yl]methyl (E)-3-(3,4-dimethoxyphenyl)prop-2-enoate
29	Phosphorylcholine
30	Thelephoric acid
31	Chrysanthellin A
32	alpha-D-Glucose
33	Aspartylhydroxyproline
34	Deoxyuridine
35	gamma-Glutamylmethionine
36	CL(8:0/12:0/12:0/15:0)
37	Methyl glucooleoside
38	L-Arginine
39	4-Methylpentyl glucosinolate
40	LysoPC(18:0/0:0)
41	Tyr-Tyr-Tyr
42	dUMP
43	Fumarprotocetraric Acid
44	N-Acetylaspartylglutamic acid
45	8-benzyl-9,10-dimethoxy-5,8-dihydro-6H-[1,3]dioxolo[4,5-g]isoquino[3,2-a]isoquinoline
46	Oleoylcarnitine
47	N6-Succinyl Adenosine
48	Rhodiocyanoside A
49	Glycyl-tyrosyl-alanine
50	2,4,5-Trihydroxy-3-[(3,4,5-trihydroxybenzoyl)oxy]benzoic acid
51	2-Hydroxyadipic acid
52	Genipin 1-gentiobioside

53	Galactosylglycerol
54	5-[(E)-2-(4-Hydroxyphenyl)vinyl]-2-[(1E)-3-methyl-1,3-butadien-1-yl]-1,3-benzenediol
55	(2R,3R,4R,6aR,6bS,8aR,14bR)-2,3,12-trihydroxy-4,6a,6b,11,11,14b-hexamethyl-8a-[(2S,3R,4S,5S,6R)-3,4,5-trihydroxy-6-(hydroxymethyl)oxan-2-yl]oxycarbonyl-1,2,3,4a,5,6,7,8,9,10,12,12a,14,14a-tetradecahydropicene-4-carboxylic acid
56	N-[(6-Hydroxy-1,3-benzodioxol-5-yl)carbonyl]glycine
57	LysoPC(16:0/0:0)
58	Tetrahydrofurfuryl acetate
59	p-Menth-1-en-4-ol
60	Indole-3-carboxaldehyde
61	Creatine
62	Pipecolic acid
63	Neocasomorphin (1-5)
64	Limonin
65	1-(2,6-Dihydroxy-4-methoxyphenyl)-3-phenyl-1-propanone
66	Erucic acid
67	2-Benzoxazolol
68	Proline betaine
69	N-Acetylglutamine
70	Ethyl aconitate
71	gamma-Glutamylthreonine
72	O-Linoleoylcarnitine
73	L-Gulose
74	Paraxanthine
75	3-Propyl-1,2-cyclopentanedione
76	L-2-Amino-4-methylenepentanedioic acid
77	IDP

78	4-Pentenyl acetate
79	LysoPC(0:0/16:0)
80	Histidine
81	Trisaccharides (Hex-Hex-AcetylHexA)
82	Arachidic acid
83	5-Methyltetrahydropteroyltri-L-glutamic acid
84	Elaidic acid
85	LysoPC(17:0/0:0)
86	Creatinine
87	1,2,3-Trihydroxybenzene
88	[(1R,5S,6S)-5-acetyloxy-3-(hydroxymethyl)-2-oxo-6-propan-2-ylcyclohex-3-en-1-yl] (E)-2-methylbut-2-enoate
89	Glutamylglycine
90	LysoPE(20:1(11Z)/0:0)
91	8-Methoxykynurenate
92	Salsolinol
93	Stearoylcarnitine
94	N-methylethanolamine phosphate

Table S5. Summary of statistical testing results for UPLC-QTOF-MS (positive mode) plasma metabolites between the HC and IBS groups. The table presents raw *p*-values (Welch's *t*-test or Mann–Whitney U test), FDR-adjusted *q*-values, and effect sizes (Hedges' *g*)

No.	Metabolite	*p*-value	*q*-value	Hedges' *g*
1	(+)-Tubotaiwine	0.9641	0.9862	-0.2469
2	(±)-3-Hydroxynonanoic acid	0.3957	0.6107	-0.5148
3	(1(10)E,4a,5E)-1(10),5-Germacradiene-12-acetoxy-4,11-diol	0.4784	0.6579	0.2415
4	(15S,16Z,17S)-16-ethylidene-4-hydroxy-15-{[(2S,3R,4S,5S,6R)-3,4,5-trihydroxy-6-(hydroxymethyl)oxan-2-yl]oxy}-2,10,14,20-tetraoxatetracyclo[21.2.2.13,7.012,17]octacosa-1(25),3(28),4,6,12,23,26-heptaene-11,19-dione	0.5804	0.7637	0.1296
5	(1alpha,2alpha,4betaH,6alpha,8R)-p-Menthane-2,6,8,9-tetrol	0.0977	0.2939	-0.5604
6	(1R,2E,7R,10E,12S,13S,15R)-12,15-dihydroxy-7-methyl-8-oxabicyclo[11.3.0]hexadeca-2,10-dien-9-one	0.0297	0.1461	0.2863
7	(1R,2R,3S,1'R)-Nepetalinic acid	0.0624	0.2320	-0.4972
8	(1S,2R,4S,9R,10R,14S,15S,17S)-9-(furan-3-yl)-1-hydroxy-15-[(1R)-1-hydroxy-2-methoxy-2-oxoethyl]-10,14,16,16-tetramethyl-7,18-dioxo-3,8-dioxapentacyclo[12.3.1.02,4.04,13.05,10]octadecan-17-yl propanoate	0.7758	0.8767	-0.2449
9	(2R,3R,4R,6aR,6bS,8aR,14bR)-2,3,12-trihydroxy-4,6a,6b,11,11,14b-hexamethyl-8a-[(2S,3R,4S,5S,6R)-3,4,5-trihydroxy-6-(hydroxymethyl)oxan-2-yl]oxycarbonyl-1,2,3,4a,5,6,7,8,9,10,12,12a,14,14a-tetradecahydropicene-4-carboxylic acid	0.0133	0.1047	-0.6737

No.	Metabolite	*p*-value	*q*-value	**Hedges' *g***
10	(2R,3R,4S,5S,6R)-2-[(2E)-4-hydroxy-3,7-dimethylocta-2,6-dienoxy]-6-(hydroxymethyl)oxane-3,4,5-triol	0.9714	0.9862	0.0096
11	(2S,3S,4S,5R,6S)-6-(Hydroxymethyl)-5-{[(2R,3S,4S,5R,6S)-3,4,5-trihydroxy-6-(hydroxymethyl)-5-{[(2R,3S,4R,5R,6S)-3,4,5-trihydroxy-6-(hydroxymethyl)tetrahydro-2H-pyran-2-yl]oxy}tetrahydro-2H-pyran-2-yl] oxy}tetrahydro-2H-pyran-2,3,4,5-tetrol	0.1005	0.2972	-0.2327
12	(2Z)-4,6-dihydroxy-2-[(4-hydroxy-3,5-dimethoxyphenyl)methylidene]-1-benzofuran-3-one	0.0018	0.0260	0.8648
13	(3E,5S,7R,8R,11E,13S,15R,16R)-3,5,7,11,13,15-hexamethyl-8,16-bis(1,3-oxazol-5-ylmethyl)-1,9-dioxacyclohexadeca-3,11-diene-2,10-dione	0.0263	0.1389	0.5491
14	(3R,5S)-1-pyrroline-3-hydroxy-5-carboxylic Acid	0.0823	0.2676	-0.4740
15	(9E)-12-hydroxyoctadec-9-enoic acid	0.1194	0.3316	0.3690
16	(9Z,11R,12S,13S,15Z)-12,13-Epoxy-11-hydroxy-9,15-octadecadienoic acid	0.3864	0.6107	0.3078
17	(E)-3-(2-Hydroxyphenyl)-2-propenal	0.2092	0.4425	-0.2857
18	(S)-Annocherine A	0.0575	0.2291	0.3247
19	(Z)-1,3-Octadiene	0.0000	0.0005	-1.3113
20	(Z)-2-octylpent-2-enedioic acid	0.9323	0.9711	0.1604
21	[(1R,5S,6S)-5-acetyloxy-3-(hydroxymethyl)-2-oxo-6-propan-2-ylcyclohex-3-en-1-yl] (E)-2-methylbut-2-enoate	0.0081	0.0814	0.6355
22	[(2R,3R,4S,5R,6R)-6-[2-(3,4-dihydroxyphenyl)ethoxy]-3,5-dihydroxy-4-[(3R,4R,5R,6S)-	0.0613	0.2313	0.5346

No.	Metabolite	*p*-value	*q*-value	Hedges' *g*
	3,4,5-trihydroxy-6-methyloxan-2-yl]oxyoxan-2-yl]methyl (E)-3-(3,4-dihydroxyphenyl)prop-2-enoate			
23	[(2R,3S,4S,5R,6R)-6-[1,7-bis(3,4-dihydroxyphenyl)-5-oxoheptan-3-yl]oxy-3,4,5-trihydroxyoxan-2-yl]methyl (E)-3-(3,4-dimethoxyphenyl)prop-2-enoate	0.0016	0.0260	0.7795
24	[(3R,3aS,4R,6R,8S,9bR)-6-acetyloxy-4-butanoyloxy-3,3a-dihydroxy-3,6,9-trimethyl-2-oxo-4,5,6a,7,8,9b-hexahydroazuleno[4,5-b]furan-8-yl] (E)-2-methylbut-2-enoate	0.0839	0.2683	0.3994
25	[2-hydroxy-3-[3,4,5-trihydroxy-6-(hydroxymethyl)oxan-2-yl]oxypropyl] (9E,12E)-octadeca-9,12-dienoate	0.5014	0.6826	0.2093
26	[5-ethenyl-3-hydroxy-4-(3-hydroxyprop-1-en-2-yl)-2-(3-methoxy-3-oxoprop-1-en-2-yl)-5-methylcyclohexyl] (Z)-4-acetyloxy-2-methylbut-2-enoate	0.4241	0.6271	-0.2535
27	[6]-Dehydrogingerdione	0.4576	0.6514	0.2377
28	1-(2,6-Dihydroxy-4-methoxyphenyl)-3-phenyl-1-propanone	0.0031	0.0375	0.7525
29	1-(3,4-Dihydro-5-(2R)-2-piperidinyl-1(2H)-pyridinyl)ethanone	0.6373	0.8003	0.1182
30	1-(Hydroxymethyl)-5,5-dimethyl-2,4-imidazolidinedione	0.1845	0.4092	-0.4079
31	1-Acetoxy-2-hydroxy-16-heptadecyn-4-one	0.2173	0.4427	0.3676
32	1-hexadecyl-glycero-3-phosphate	0.3867	0.6107	0.1158
33	1-Methylpseudouridine	0.3950	0.6107	-0.4055
34	1-Oleoylglycerophosphoserine	0.2563	0.4731	0.3768
35	1-phosphatidyl-1D-myo-inositol 3-phosphate	0.3218	0.5497	-0.1980
36	1-Stearoylglycerophosphoserine	0.2118	0.4427	0.4187

No.	Metabolite	*p*-value	*q*-value	Hedges' *g*
37	1,2,3-Trihydroxybenzene	0.0030	0.0375	-0.6497
38	11,12-Dimethoxydihydrokawain	0.6596	0.8098	-0.2683
39	12-Methyltridecanal	0.2501	0.4703	-0.3080
40	16-Hydroxyhexadecanoic acid	0.1555	0.3684	0.0974
41	2'-O-Methyl-5-methylcytidine	0.0079	0.0814	0.7190
42	2'-O-Methylcytidine	0.3442	0.5808	0.2758
43	2-[3-[(E)-6-acetyloxy-2-hydroxy-6-methyl-3-oxohept-4-en-2-yl]-6-(carboxymethyl)-2-hydroxy-3a,5a,9b-trimethyl-5-oxo-2,3,4,6,9,9a-hexahydro-1H-cyclopenta[a]naphthalen-7-yl]-2-methylpropanoic acid	0.0796	0.2639	-0.1241
44	2-Aminobenzoic acid	0.8213	0.8963	0.0787
45	2-Benzoxazolol	0.2387	0.4689	-0.3061
46	2-Hexadecanone	0.7573	0.8641	-0.0401
47	2-Hydroxy-3-phenylbutanoic acid	0.2489	0.4703	-0.3732
48	2-Hydroxy-4-methoxy-3,5-bis(3-methylbut-2-enyl)-6-pentylbenzoic acid	0.3733	0.6075	0.0688
49	2-Hydroxyadipic acid	0.0086	0.0814	-0.6808
50	2-Hydroxybenzaldehyde	0.8203	0.8963	-0.0082
51	2-Hydroxystearic acid	0.3032	0.5415	0.2827
52	2-Isopropylphenyl methylcarbamate	0.0017	0.0260	0.6933
53	2-Methyl-5-nitroaniline	0.6119	0.7769	-0.2029
54	2-Naphthaleneacetic acid, decahydro-1-hydroxy-4a-methyl-alpha,8-bis(methylene)-, (1S-(1alpha,2beta,4abeta,8aalpha))-	0.0702	0.2442	0.2459
55	2-Octenoylcarnitine	0.6920	0.8167	0.1113
56	2-Phenoxyethyl isobutyrate	0.1549	0.3684	-0.2400
57	2-Phenylglycine	0.3940	0.6107	-0.2547
58	2-Pyrroloylglycine	0.2260	0.4571	-0.3269
59	2,3-Undecanedione	0.1551	0.3684	0.4517
60	2,3,4-Trimethylhexane	0.7020	0.8250	0.1165

No.	Metabolite	*p*-value	*q*-value	Hedges' *g*
61	2,4,5-Trihydroxy-3-[(3,4,5-trihydroxybenzoyl)oxy]benzoic acid	0.0479	0.1997	-0.6298
62	3-(4-Fluorobenzoyl)propionic acid	0.6655	0.8098	0.0432
63	3-[(2-Isopropyl-5-methylcyclohexyl)oxy]-2-methyl-1,2-propanediol	0.2330	0.4644	0.1936
64	3-[4-methyl-1-(2-methylpropanoyl)-3-oxocyclohexyl]butanoic acid	0.5184	0.6920	0.1259
65	3-Amino-4-hydroxybenzoic acid	0.2418	0.4703	-0.3249
66	3-Hexenedioic acid	0.0323	0.1557	-0.3334
67	3-Methyl sulfolene	0.4097	0.6244	-0.0226
68	3-Polyprenyl-4-hydroxy-5-methoxybenzoate	0.3968	0.6107	-0.3071
69	3-Propyl-1,2-cyclopentanedione	0.4186	0.6244	-0.2545
70	4-(1-Hydroxy-2-propanyl)-1-methyl-1,2-cyclohexanediol	0.1109	0.3145	0.4474
71	4-[(2R,5R)-3-hydroxy-5-[4-[2-(4-hydroxyphenyl)ethylamino]-4-oxobutanoyl]oxy-6-methyloxan-2-yl]oxypentanoic acid	0.0670	0.2423	0.4318
72	4-Carboxy-3-methoxy-2,5,6-trimethylphenyl 4-((2,4-dihydroxy-3,6-dimethylbenzoyl)oxy)-2-methoxy-3,5,6-trimethylbenzoate	0.0707	0.2442	0.3886
73	4-Hydroxymethamphetamine	0.7779	0.8767	-0.2496
74	4-Methyl-2-pentenoic acid	0.0000	0.0013	-1.3030
75	4-Methylpentyl glucosinolate	0.1676	0.3810	-0.6088
76	4-Pentenyl acetate	0.0248	0.1366	-0.4597
77	5'-Methylthioadenosine	0.8452	0.9151	-0.1674
78	5',5''',8,8''-Tetrahydroxy-3',3''',4',4''',7',7''-hexamethoxy-5,5''-biflavan	0.0002	0.0062	1.0343
79	5-[(E)-2-(4-Hydroxyphenyl)vinyl]-2-[(1E)-3-methyl-1,3-butadien-1-yl]-1,3-benzenediol	0.4618	0.6514	-0.1569

No.	Metabolite	*p*-value	*q*-value	Hedges' *g*
80	5-[(Z)-14-(3,5-dihydroxyphenyl)tetradec-10-enyl]benzene-1,3-diol	0.6497	0.8058	0.0494
81	5-[1,2,4a-trimethyl-5-(propanoyloxymethyl)-2,3,4,7,8,8a-hexahydronaphthalen-1-yl]-3-methylpentanoic acid	0.1175	0.3298	0.3738
82	5-Methylcytidine	0.4447	0.6460	-0.1746
83	5-Nonyloxytryptamine	0.1417	0.3604	-0.1458
84	5,6,7,8-Tetrahydro-4-methylquinoline	0.6537	0.8061	-0.2525
85	6-(furan-3-yl)-6,8,12,16,21-pentahydroxy-7,15-dimethyl-9-oxo-3,17,19-trioxaheptacyclo[9.9.3.01,10.02,4.02,7.011,18.015,23]tricosan-14-yl 2-methylbutanoate	0.6754	0.8147	-0.1385
86	6-Hydroxy-3,6,9-trimethyl-2-oxo-2,3,3a,4,5,6,8,9b-octahydroazuleno[4,5-b]furan-4-yl acetate	0.5617	0.7462	0.2524
87	6alpha-Hydroxy-hydromorphone	0.8137	0.8963	-0.1912
88	7-Hydroxypestalotin	0.8213	0.8963	0.2703
89	8-Methoxykynurenate	0.0361	0.1654	-0.5917
90	8,11-Heptadecadienal	0.4128	0.6244	0.1945
91	9-(Methoxycarbonyl)-9-decenoic acid	0.2800	0.5100	0.1437
92	9-Decenoylcarnitine	0.6131	0.7769	0.1929
93	Acetylbalchanolide	0.9287	0.9711	-0.0246
94	Alanylleucine	0.2019	0.4328	-0.3560
95	Allopurinol	0.8872	0.9420	0.0623
96	Allopurinol riboside	0.3162	0.5497	-0.3197
97	alpha-D-Glucose	0.0003	0.0081	-0.8702
98	alpha-Linolenic acid	0.5918	0.7657	-0.1304
99	alpha-Micropteroxanthin B	0.4637	0.6514	0.3961
100	Amifloxacin	0.7644	0.8687	0.0921
101	Aminocaproic acid	0.3534	0.5910	-0.1953
102	Anserine	0.8637	0.9241	-0.0558

No.	Metabolite	*p*-value	*q*-value	Hedges' *g*
103	ar-Artemisene	0.0153	0.1050	0.3824
104	Arachidic acid	0.4471	0.6460	-0.2119
105	Asparaginyl-Valine	0.0184	0.1150	-0.5532
106	Benzidine	0.0001	0.0039	-1.1533
107	Betaine	0.0010	0.0248	-0.8586
108	Biliverdin	0.1606	0.3711	0.1355
109	Bufalin	0.7232	0.8413	-0.1602
110	Buprenorphine	0.8746	0.9322	0.0521
111	Caffeine	0.6059	0.7769	0.0392
112	Carapin	0.2826	0.5112	0.2822
113	Celastrol	0.0516	0.2117	0.4539
114	Cer[NS] d34:2	0.0000	0.0024	1.0080
115	Cer[NS] d45:1	0.0148	0.1050	0.4913
116	Chaetoglobosin N	0.9754	0.9862	-0.2606
117	Chrysanthellin A	0.0256	0.1380	0.7335
118	Chrysin-7-O-glucuronide	0.6840	0.8152	-0.1075
119	CL(8:0/12:0/12:0/15:0)	0.0103	0.0913	0.7628
120	Clovanediol Diacetate	0.2489	0.4703	0.4666
121	Coumarin	0.4201	0.6244	-0.0518
122	Creatine	0.0018	0.0260	-0.7655
123	Creatinine	0.0270	0.1401	-0.6419
124	Curvulin	0.9559	0.9862	-0.2245
125	Cyclandelate	0.0767	0.2606	0.3126
126	Decanoylcarnitine	0.8120	0.8963	0.1660
127	Dehydro-piliformic-acid	0.0012	0.0248	-1.0173
128	Dehydrocurvularin	0.3975	0.6107	-0.3442
129	Denatonium_TP222	0.0198	0.1213	0.3137
130	Deoxyadenosine monophosphate	0.0000	0.0019	1.2071
131	Deoxyribose 5-monophosphate	0.3616	0.5926	-0.2752
132	Dexchlorpheniramine	0.7065	0.8268	-0.0514
133	Didanosine	0.5045	0.6833	-0.4332
134	Dihydroergocristine	0.1345	0.3524	0.4044

No.	Metabolite	*p*-value	*q*-value	Hedges' *g*
135	Dihydroisomorphine-6-glucuronide	0.9828	0.9900	-0.1754
136	Dimethachlor OXA	0.0148	0.1050	-0.4442
137	Dipropyl phthalate	0.0222	0.1297	-0.4295
138	Dodecanoic acid	0.0031	0.0375	0.6549
139	Elaidic acid	0.0376	0.1663	0.5017
140	Ellagic acid	0.8360	0.9087	-0.0908
141	Erucic acid	0.0423	0.1816	0.5230
142	Erysopine	0.9737	0.9862	0.2817
143	Ethyl aconitate	0.0367	0.1654	-0.5429
144	Fagomine	0.0175	0.1121	-0.6015
145	FAHFA(16:1(9Z)/9-O-18:0)	0.1455	0.3604	0.3046
146	fluvoxamino acid	0.1057	0.3059	0.1933
147	Fumarprotocetraric Acid	0.0711	0.2442	-0.7265
148	Furofoline	0.6087	0.7769	-0.3735
149	Galactosylglycerol	0.1108	0.3145	-0.5943
150	gamma-Glutamylmethionine	0.0102	0.0913	-0.8221
151	gamma-Glutamylthreonine	0.1289	0.3441	-0.6827
152	Genipin 1-gentiobioside	0.0959	0.2930	-0.6205
153	Gingerol	0.2588	0.4745	0.2836
154	Glucosylgalactosyl hydroxylysine	0.4135	0.6244	0.3159
155	Glutamylglycine	0.0062	0.0685	0.6536
156	Glutarylcarnitine	0.6434	0.8042	0.0630
157	Glyceryl linolenate	0.4550	0.6514	0.2636
158	Glycocholic acid	0.5878	0.7657	0.0259
159	Glycyl-tyrosyl-alanine	0.0242	0.1360	0.6667
160	Heptadecanoic acid	0.9938	0.9974	0.0109
161	Hippuric acid	0.0365	0.1654	0.3755
162	Hirsuteine	0.5153	0.6913	0.2405
163	Histidine	0.0149	0.1050	-0.6164
164	Homarine	0.8947	0.9426	0.1343
165	Hypoxanthine	0.4671	0.6519	-0.1888
166	Indole-3-carboxaldehyde	0.0028	0.0375	0.7641

No.	Metabolite	*p*-value	*q*-value	Hedges' *g*
167	Indoleacrylic acid	0.1790	0.4034	-0.3456
168	Isoleucine	0.9132	0.9586	0.1180
169	Isoleucyl-Glutamate	0.3961	0.6107	0.1340
170	Isopersin	0.0680	0.2428	0.5291
171	Isoproterenol	0.0288	0.1455	-0.4722
172	Kaempferol 3-glucuronide	0.3547	0.5910	-0.1744
173	Kynurenine	0.6188	0.7805	0.1245
174	L-2-Amino-4-methylenepentanedioic acid	0.0614	0.2313	-0.6740
175	L-2,3-Dihydrodipicolinate	0.5872	0.7657	-0.4922
176	L-Acetylcarnitine	0.0608	0.2313	-0.3709
177	L-Arginine	0.2439	0.4703	-0.2895
178	L-Carnitine	0.0365	0.1654	-0.5013
179	L-Homoserine	0.3402	0.5775	-0.2690
180	L-Norleucine	0.7250	0.8413	0.1780
181	L-Tyrosine	0.1585	0.3694	-0.4184
182	Lansimide 3	0.1804	0.4034	0.3253
183	Licoagroside B	0.4723	0.6527	-0.3135
184	Limonin	0.3568	0.5910	-0.5010
185	LysoPC(0:0/18:0)	0.8190	0.8963	0.0170
186	LysoPC(0:0/18:2(9Z,12Z))	0.7933	0.8905	-0.0548
187	LysoPC(15:0/0:0)	0.9980	0.9980	0.0467
188	LysoPC(16:1/0:0)	0.3870	0.6107	0.2288
189	LysoPC(18:1/0:0)	0.2153	0.4427	0.3661
190	LysoPC(18:3(9Z,12Z,15Z)/0:0)	0.4643	0.6514	0.1094
191	LysoPC(20:3(8Z,11Z,14Z)/0:0)	0.8516	0.9181	-0.0400
192	LysoPC(22:6(4Z,7Z,10Z,13Z,16Z,19Z)/0:0)	0.7455	0.8549	-0.1041
193	LysoPE(0:0/18:0)	0.8049	0.8963	0.0810
194	LysoPE(0:0/20:0)	0.2946	0.5296	0.3026
195	LysoPE(0:0/22:5(4Z,7Z,10Z,13Z,16Z))	0.1399	0.3604	0.2748
196	LysoPE(0:0/22:6(4Z,7Z,10Z,13Z,16Z,19Z))	0.6856	0.8152	0.1180

No.	Metabolite	*p*-value	*q*-value	Hedges' *g*
197	LysoPE(16:0/0:0)	0.6655	0.8098	-0.1531
198	LysoPE(18:2(9Z,12Z)/0:0)	0.0085	0.0814	0.6196
199	LysoPE(20:1(11Z)/0:0)	0.2325	0.4644	0.3238
200	m-Coumaric acid	0.2030	0.4328	-0.3877
201	Mecamylamine	0.4486	0.6460	-0.1157
202	methyl (Z)-5-[(1R,4aR,8aR)-5,5,8a-trimethyl-2-methylidene-3,4,4a,6,7,8-hexahydro-1H-naphthalen-1-yl]-3-(acetyloxymethyl)pent-2-enoate	0.1018	0.2978	0.3385
203	Methyl glucooleoside	0.0017	0.0260	0.8632
204	Methylephedrine	0.8932	0.9426	0.0632
205	Metipranolol	0.5069	0.6833	0.2184
206	Metolachlor OA	0.0827	0.2676	0.1610
207	Montelukast	0.3215	0.5497	-0.2548
208	Myristic acid	0.0983	0.2939	0.4112
209	Myrsinone	0.1912	0.4174	0.2600
210	N-(1-Deoxy-b-D-fructopyranosyl) (R)C(S)S-alliin	0.1567	0.3684	-0.3269
211	N-[(6-Hydroxy-1,3-benzodioxol-5-yl)carbonyl]glycine	0.0381	0.1663	-0.6978
212	N-[2-Hydroxy-2-(4-hydroxyphenyl)ethyl]cinnamide	0.2452	0.4703	0.3743
213	N-Acetylaspartylglutamic acid	0.0014	0.0260	0.7983
214	N-Acetylglutamine	0.0911	0.2880	-0.7127
215	N-Acetylproline	0.4693	0.6519	0.2533
216	N-Cyano-N'-(1,1-dimethylpropyl)-N''-(3-pyridinyl)guanidine	0.2562	0.4731	0.2186
217	N-Glycolylneuraminic acid	0.6505	0.8058	-0.2526
218	N-methylethanolamine phosphate	0.2171	0.4427	-0.2671
219	N-Myristoylethanolamine	0.5931	0.7657	-0.1820
220	N,N,N-Trimethylethenaminium	0.4177	0.6244	-0.2422
221	N6-Succinyl Adenosine	0.3620	0.5926	-0.5540
222	Neocasomorphin (1-5)	0.0168	0.1118	0.6457
223	Nicotine	0.4476	0.6460	-0.2096

No.	Metabolite	*p*-value	*q*-value	Hedges' *g*
224	Nicotinic acid mononucleotide	0.8546	0.9181	0.0331
225	Nitrazepam	0.1455	0.3604	-0.4185
226	Norvaline	0.0242	0.1360	-0.5461
227	O-Linoleoylcarnitine	0.0930	0.2899	0.4715
228	Octanoylcarnitine	0.9428	0.9784	0.1357
229	Oleoylcarnitine	0.0125	0.1012	0.7226
230	Oxamniquine	0.3185	0.5497	0.2724
231	Oxfendazole	0.3215	0.5497	0.2548
232	p-Menth-1-en-4-ol	0.0041	0.0468	0.6175
233	PA(10:0/13:0)	0.1261	0.3399	0.3896
234	Palmitoylcarnitine	0.4454	0.6460	0.2468
235	Paraxanthine	0.0216	0.1294	-0.6249
236	Paromomycin	0.6732	0.8147	0.0160
237	Perfluorotridecanoic acid	0.0118	0.0980	0.4930
238	Phenylacetylglutamine	0.2141	0.4427	0.0148
239	Phenylalanine	0.7361	0.8505	0.0938
240	Phenylephrine	0.9618	0.9862	0.0370
241	Phosphorylcholine	0.0001	0.0051	0.8308
242	Pipecolic acid	0.0109	0.0933	-0.7292
243	Piperine	0.7461	0.8549	0.0511
244	Piroxicam	0.3215	0.5497	0.2548
245	Pristanic acid	0.1448	0.3604	0.3339
246	Proglobeflowery acid	0.0441	0.1866	0.2465
247	Proline	0.1624	0.3722	-0.3753
248	Proline betaine	0.0591	0.2313	-0.6304
249	Pseudouridine	0.4984	0.6819	0.1090
250	Pyridoxamine	0.0668	0.2423	-0.4753
251	Pyruvic acid	0.3937	0.6107	-0.0467
252	Rhodiocyanoside A	0.0171	0.1118	0.6199
253	Rosoxacin	0.5689	0.7521	-0.2783
254	S4:16(P3:14/F1:2)	0.0002	0.0062	0.9910
255	Salsolinol	0.0793	0.2639	0.4067
256	Serotonin	0.1241	0.3399	0.6077

No.	Metabolite	*p*-value	*q*-value	Hedges' *g*
257	Sotalol	0.6878	0.8152	-0.2881
258	Specnuezhenide	0.0006	0.0155	0.9673
259	Sphaerindicin	0.0011	0.0248	0.9034
260	Sphinganine	0.3122	0.5497	-0.2380
261	Sphingosine 1-phosphate	0.1435	0.3604	0.3756
262	Stearoylcarnitine	0.2514	0.4703	0.3362
263	Tanacetol A	0.0567	0.2291	0.2571
264	Telmisartan	0.6847	0.8152	-0.2545
265	Tetrahydrofurfuryl acetate	0.0291	0.1455	-0.7792
266	Theophylline	0.1259	0.3399	0.2057
267	Trismethoxyresveratrol	0.9684	0.9862	0.0046
268	Tryptophyl-Arginine	0.1999	0.4328	0.3770
269	Ureidopropionic acid	0.2356	0.4662	0.0719
270	Urocanic acid	0.0150	0.1050	0.2771
271	Ursocholic acid	0.1331	0.3519	0.0682
272	Valdecoxib	0.0938	0.2899	-0.5916
273	Valerylcarnitine	0.1545	0.3684	-0.3644
274	Verapamil	0.1492	0.3662	0.1756
275	Voglibose	0.1874	0.4122	0.4101

Table S6. Summary of statistical testing results for UPLC-QTOF-MS (negative mode) plasma metabolites between the HC and IBS groups. The table presents raw *p*-values (Welch's *t*-test or Mann–Whitney U test), FDR-adjusted *q*-values, and effect sizes (Hedges' *g*)

No.	Metabolite	*p*-value	*q*-value	Hedges' *g*
1	(2R,3R,4R,5R,6S)-2-[[(2R,3S,4S,5R,6S)-6-(4-ethenylphenoxy)-3,4,5-trihydroxyoxan-2-yl]methoxy]-6-methyloxane-3,4,5-triol	0.6553	0.9112	0.1336
2	(2S)-8-[(E)-3-hydroxy-3-methylbut-1-enyl]-5,7-dimethoxy-2-phenyl-2,3-dihydrochromen-4-one	0.9936	0.9972	0.0418
3	(3-Methyl-2-butenyl)-benzene	0.6607	0.9112	0.2487
4	(3S,4S)-5-[(3S,4S)-4,10-dihydroxy-7,9-dimethoxy-3-methyl-3,4-dihydro-1H-benzo[g]isochromen-5-yl]-7,9-dimethoxy-3-methyl-3,4-dihydro-1H-benzo[g]isochromene-4,10-diol	0.0462	0.3748	0.5088
5	(E)-3-(4-methoxyphenyl)-1-[2,4,6-trimethoxy-3-(3-methylbut-2-enyl)phenyl]prop-2-en-1-one	0.4034	0.7664	0.0357
6	[(2R,3R,4S,5R,6S)-2-(hydroxymethyl)-4,5,6-tris[(3,4,5-trihydroxybenzoyl)oxy]oxan-3-yl] 3,4,5-trihydroxybenzoate	0.9112	0.9919	-0.0706
7	[(2R,3S,4S,5R,6R)-6-[1,7-bis(4-hydroxyphenyl)-5-oxoheptan-3-yl]oxy-3,4,5-trihydroxyoxan-2-yl]methyl (E)-3-(3,4-dimethoxyphenyl)prop-2-enoate	0.0084	0.1230	0.7466

No.	Metabolite	*p*-value	*q*-value	Hedges' *g*
8	[(4E)-7-acetyloxy-6-hydroxy-2-methyl-10-oxo-2,3,6,7,8,9-hexahydrooxecin-3-yl] (E)-but-2-enoate	0.9490	0.9919	-0.1654
9	[6-[4-Acetyloxy-3-hydroxy-2,5-bis(hydroxymethyl)oxolan-2-yl]oxy-2-(hydroxymethyl)-4,5-di(pentanoyloxy)oxan-3-yl] pentanoate	0.1714	0.5153	0.3691
10	[8,8-dimethyl-9-(3-methylbut-2-enoyloxy)-2-oxo-9,10-dihydropyrano[2,3-f]chromen-10-yl] 3-methylbut-2-enoate	0.5972	0.9082	0.0023
11	1-(2-Deoxy-α-D-erythro-pentofuranosyl)-2,4-dioxo-1,2,3,4-tetrahydro-5-pyrimidinecarboxylic acid	0.1100	0.4350	0.3585
12	1-(3,4-Dihydroxyphenyl)-7-(4-hydroxyphenyl)-3,5-heptanedione	0.2836	0.6371	0.3817
13	1,1,1-Trifluoro-3-methylbutan-2-one	0.8142	0.9586	0.2294
14	1,3-Dihydro-(2H)-indol-2-one	0.0598	0.4030	0.3858
15	1,7,7-trimethylbicyclo[2.2.1]hept-2-yl 4-hydroxy-3-methoxybenzoate	0.4927	0.8251	-0.0673
16	11,12-Methylenedioxykopsinaline	0.9116	0.9919	0.1138
17	12,13-DHOME	0.4222	0.7706	0.3957
18	17:0 Lyso PE-d5	0.3241	0.6760	0.2233
19	2'-Hydroxy-4',6'-dimethoxy-3'-methylacetophenone	0.7598	0.9485	0.0583
20	2-(2-hydroxybut-3-en-2-yl)-3a,6,6,9a-	0.0690	0.4030	0.1439

No.	Metabolite	*p*-value	*q*-value	Hedges' *g*
	tetramethyl-2,4,5,5a,7,8,9,9b-octahydro-1H-benzo[e][1]benzofuran-4,5-diol			
21	2-(3-Carboxy-3-aminopropyl)-L-histidine	0.4973	0.8251	-0.0685
22	2-beta-D-Glucopyranosyl-1,3,6-trihydroxy-5-methoxy-9H-xanthen-9-one	0.9412	0.9919	-0.0654
23	2-Hydroxyacetophenone sulfate	0.1792	0.5232	0.3338
24	2-Methyl-3-hydroxybutyric acid	0.9972	0.9972	0.1765
25	2-Methylglutaric Acid	0.2011	0.5384	0.3263
26	2-Thiocytidine	0.2681	0.6214	0.3665
27	2-Tridecenal	0.8282	0.9673	0.0662
28	2,3-Dihydroxy-2,4-cyclopentadien-1-one	0.7282	0.9313	0.1118
29	2,3-Dihydroxyvaleric acid	0.2661	0.6214	-0.0144
30	3-[(2R,3S)-5-hydroxy-2,3,8,8-tetramethyl-4-oxo-2,3-dihydropyrano[2,3-h]chromen-6-yl]hexanoic acid	0.1208	0.4408	0.3145
31	3-[2-(6,7-dihydroxy-1,2,4a-trimethylspiro[3,4,6,7,8,8a-hexahydro-2H-naphthalene-5,2'-oxirane]-1-yl)ethyl]-2-hydroxy-2H-furan-5-one	0.4864	0.8251	0.3996
32	3-[3-(Sulfooxy)phenyl]propanoic acid	0.1576	0.5035	-0.0466
33	3-{[(7-methoxy-2-oxochromen-4-yl)methyl]methylamino}propanenitrile	0.0773	0.4030	0.2092
34	3-Hydroxyibuprofen	0.0732	0.4030	-0.2773

No.	Metabolite	*p*-value	*q*-value	Hedges' *g*
35	3-Methoxyphenol sulfate	0.9277	0.9919	-0.1918
36	3-Pyridylacetic acid	0.8049	0.9586	0.1270
37	3Alpha-Hydroxy-3-Deoxyangolensic Acid Methyl Ester	0.7193	0.9313	-0.0763
38	4-[3-[(2E)-3,7-dimethylocta-2,6-dienyl]-2,6-dihydroxybenzoyl]-3-formyl-5-hydroxybenzoic acid	0.7313	0.9313	0.1625
39	4-Hydroxy-2-methoxy-3-(3-methylbut-2-enyl)benzoic acid	0.7336	0.9313	0.2453
40	4-Hydroxybenzoic acid	0.9716	0.9919	0.0983
41	4-Methoxy-6-[2-[4-(3-methylbut-2-enoxy)phenyl]ethyl]-1,3-benzodioxole	0.4427	0.7936	-0.0572
42	4-Thiouridine	0.1248	0.4444	-0.4132
43	5-(12,15-Heneicosadienyl)-1,3-benzenediol	0.1453	0.4934	0.3171
44	5-(4-hydroxyphenyl)pentanoic Acid	0.6369	0.9112	-0.2465
45	5-Acetylamino-6-formylamino-3-methyluracil	0.6204	0.9112	0.1209
46	5-Hydroxy-1-isopropyl-4-methyl-3-cyclohexen-1-yl hexopyranoside	0.2481	0.6214	0.3158
47	5-Methyltetrahydropteroyltri-L-glutamic acid	0.5397	0.8659	0.1479
48	6,7-Diketolithocholic acid	0.8529	0.9729	0.1270
49	7-Ketocholesterol	0.0431	0.3712	0.4714
50	8-benzyl-9,10-dimethoxy-5,8-dihydro-6H-[1,3]dioxolo[4,5-	0.0286	0.2987	-0.7146

No.	Metabolite	*p*-value	*q*-value	Hedges' *g*
	g]isoquino[3,2-a]isoquinoline			
51	9-[(3,7-Dimethyl-2,6-octadienyl)oxy]-7H-furo[3,2-g][1]benzopyran-7-one	0.6743	0.9112	-0.1861
52	9-phenyl-1-(2,4,6-trihydroxyphenyl)nonan-1-one	0.1071	0.4350	0.2096
53	Abietic Acid	0.6189	0.9112	-0.0994
54	Acetaminophen glucuronide	0.9580	0.9919	-0.2576
55	Acetyl citrate	0.4558	0.8018	-0.2095
56	Aconitic acid	0.4794	0.8251	-0.0268
57	Aldosterone	0.5889	0.9082	0.1362
58	Apramycin	0.0911	0.4158	0.2113
59	Aspartylhydroxyproline	0.0050	0.0908	-0.8338
60	Baccatin III	0.3136	0.6760	0.1551
61	Biflavonoid-flavone base + 3O and flavone base + 3O + 1Prenyl	0.1507	0.5001	0.2466
62	Bilirubin	0.6210	0.9112	0.0066
63	Cephalomannine	0.2925	0.6471	-0.2133
64	Chalepin acetate	0.0144	0.1910	0.5370
65	Chenodeoxycholic acid	0.1110	0.4350	0.1165
66	Chenodeoxycholic acid glycine conjugate	0.2724	0.6214	0.1670
67	Cholic acid	0.1918	0.5343	0.1480
68	Citric acid	0.4161	0.7690	-0.0797
69	Cortisol	0.9639	0.9919	-0.0538
70	D-4-Hydroxy-2-oxoglutarate	0.8465	0.9729	0.1663
71	D-Glutamine	0.3359	0.6908	-0.3309
72	D-Mannose	0.2028	0.5384	-0.2333
73	Delphinidin 3-galactoside	0.0768	0.4030	0.3793
74	Deoxycholic acid	0.7881	0.9533	-0.1442
75	Deoxyuridine	0.0036	0.0754	-0.8585

No.	Metabolite	*p*-value	*q*-value	Hedges' *g*
76	Dexrazoxane	0.2284	0.5851	0.1253
77	Dicaffeoyl Coumaroyl Spermidine	0.0009	0.0312	0.9402
78	Diethylphosphate	0.0988	0.4350	0.2810
79	Dihydroflavonol + 2O, 2Prenyl	0.1048	0.4350	0.3557
80	Dihydrogedunic acid, methyl ester	0.1940	0.5343	0.0973
81	Dihydroplumericinic acid	0.6402	0.9112	-0.1089
82	Docosahexaenoic Acid-d5	0.3431	0.6958	-0.1151
83	dUMP	0.0343	0.3334	-0.5322
84	Ecabet	0.7783	0.9533	-0.0279
85	Ethyl acetoacetate	0.6732	0.9112	0.2426
86	Ethyl glucuronide	0.1449	0.4934	-0.4016
87	Feruloylquinic acid	0.1177	0.4407	-0.2324
88	Furanone A	0.0805	0.4053	0.3292
89	gamma-Glutamylhistidine	0.3788	0.7373	-0.3303
90	Gibberellin A61	0.8830	0.9841	-0.0075
91	Ginsenoside Rb1	0.0007	0.0312	0.9075
92	Glucosidetathione	0.3165	0.6760	0.2601
93	Glutamylaspartic acid	0.7053	0.9277	-0.2396
94	Hypoletin 8-gentiobioside	0.8598	0.9731	0.1044
95	IDP	0.4457	0.7936	0.1865
96	Indolelactic acid	0.7793	0.9533	0.1226
97	Indoxyl sulfate	0.9889	0.9972	0.0834
98	Inosine	0.4042	0.7664	-0.3827
99	Inosine 2',3'-cyclic phosphate	0.0015	0.0445	-0.8809
100	I(-)	0.0084	0.1230	NA
101	Ketoleucine	0.6681	0.9112	0.1941
102	L-Gulose	0.0601	0.4030	0.4221
103	Linezolid	0.9691	0.9919	0.1881
104	Lithocholic acid glycine conjugate	0.7900	0.9533	0.1738

No.	Metabolite	*p*-value	*q*-value	Hedges' *g*
105	LPS 15:0-d5	0.2644	0.6214	0.0996
106	LysoPC(0:0/16:0)	0.8515	0.9729	-0.0230
107	LysoPC(10:0/0:0)	0.5625	0.8926	0.2161
108	LysoPC(16:0/0:0)	0.1729	0.5153	0.3664
109	LysoPC(17:0/0:0)	0.1593	0.5035	0.3728
110	LysoPC(18:0/0:0)	0.2240	0.5839	0.3634
111	LysoPC(18:2/0:0)?	0.4132	0.7690	0.2309
112	LysoPE(0:0/16:0)	0.6924	0.9190	0.0744
113	Mammeigin	0.3199	0.6760	-0.1204
114	Mesulfenfos	0.5091	0.8259	0.0058
115	Methyl (9Z)-6'-oxo-6,5'-diapo-6-carotenoate	0.9512	0.9919	0.2292
116	Mimosine	0.6803	0.9112	0.1055
117	Monoelaidin	0.4973	0.8251	0.4593
118	N-[(1-Oxo-1H-isochromen-3-yl)carbonyl]glycine	0.1621	0.5035	0.4106
119	N-Acetylcarnosine	0.1831	0.5242	0.1985
120	N-Fructoryl cysteinylalanine + C2H5S	0.2562	0.6214	-0.3931
121	N-Fructosyl glutamylphenylalanine	0.7601	0.9485	-0.2080
122	N6-Threonylcarbamoyladenosine	0.8976	0.9919	0.2423
123	Nizatidine	0.0624	0.4030	0.3175
124	Oxalacetic acid	0.3512	0.6996	0.2820
125	Oxytetracycline	0.6762	0.9112	0.1774
126	p-Cresol sulfate	0.9317	0.9919	-0.1778
127	Pangamic acid	0.0262	0.2941	0.3463
128	Paracetamol sulfate	0.0252	0.2941	0.5206
129	Perillyl aldehyde	0.8670	0.9737	-0.2371
130	Phenol sulphate	0.5762	0.9046	-0.0206
131	Phomalone	0.6709	0.9112	0.1723
132	Plumbagin	0.0649	0.4030	-0.4256
133	Pyroglutamic acid	0.0432	0.3712	0.3341

No.	Metabolite	*p*-value	*q*-value	Hedges' *g*
134	Suffruticoside E	0.3546	0.6996	0.0943
135	Sulfo jasmonate	0.1132	0.4350	-0.2535
136	Thelephoric acid	0.0026	0.0638	-0.8116
137	Threonic acid	0.8094	0.9586	0.2132
138	Thymidine-3',5'-cyclic monophosphate	0.0682	0.4030	-0.3902
139	Topotecan	0.5927	0.9082	0.2568
140	Trehalose-6-phosphate	0.2549	0.6214	0.3627
141	Trifluoroacetic acid	0.0000	0.0024	-1.1793
142	Trisaccharides (Hex-Hex-AcetylHexA)	0.0516	0.3961	-0.6014
143	Tryptophan	0.5051	0.8259	0.1869
144	Tyr-Tyr-Tyr	0.0007	0.0312	0.8413
145	Uric acid	0.0876	0.4127	-0.5005
146	Uridine	0.0839	0.4086	-0.4900

Table S7. Summary of statistical testing results for GC-MS plasma metabolites between the HC and IBS groups. The table presents raw *p*-values (Welch's *t*-test or Mann–Whitney U test), FDR-adjusted *q*-values, and effect sizes (Hedges' *g*)

No.	Metabolite	*p*-value	*q*-value	Hedges' *g*
1	1,4-Benzenedicarboxylic acid	0.0836	0.4870	0.4489
2	1,5-Anhydroglucitol	0.0207	0.3813	0.5030
3	2-Hydroxybutyrate	0.4853	0.7729	-0.0815
4	Alanine	0.2478	0.6974	0.2624
5	Benzoic acid	0.0221	0.3813	0.5995
6	Cholesterol	0.9085	0.9312	-0.0294
7	Citric acid	0.3568	0.6974	0.2372
8	Elaidic acid	0.2586	0.6974	-0.0328
9	Ethanolamine	0.8631	0.9312	0.2304
10	Fructose	0.8172	0.9312	-0.2238
11	Galactose	0.1091	0.5213	-0.2876
12	Glucose	0.1635	0.6314	-0.3155
13	Glutamine	0.2503	0.6974	0.2960
14	Glycine	0.8315	0.9312	0.1740
15	Histidine	0.7274	0.9312	-0.0748
16	Hydroxybutyric acid	0.3354	0.6974	0.0330
17	Hydroxylamine	0.9357	0.9357	-0.2824
18	Hypoxanthine	0.9095	0.9312	0.4109
19	Isoleucine	0.0532	0.3813	0.4302
20	Lactic acid	0.5133	0.7819	0.2013
21	Leucine	0.0479	0.3813	0.5071
22	Lysine	0.3208	0.6974	0.2792
23	Methylamine	0.3429	0.6974	0.1126
24	Myristic acid	0.3048	0.6974	0.3333
25	Phenylalanine	0.1762	0.6314	0.2984
26	Phosphate	0.8430	0.9312	0.0877
27	Palmitic acid	0.4578	0.7571	-0.1907
28	Proline	0.0906	0.4870	0.4855
29	Propyleneglycol	0.7663	0.9312	0.0213
30	Pyroglutamic acid	0.6973	0.9312	0.0275

No.	Metabolite	*p*-value	*q*-value	Hedges' *g*
31	Pyruvate	0.5946	0.8523	0.0065
32	Serine	0.0385	0.3813	0.5405
33	Sorbose	0.8315	0.9312	-0.2442
34	Stearic acid	0.4290	0.7571	-0.2032
35	Sucrose	0.1646	0.6314	0.3355
36	Threitol	0.8805	0.9312	0.2277
37	Threonine	0.0400	0.3813	0.5041
38	Tryptophan	0.6865	0.9312	0.2539
39	Tyrosine	0.3066	0.6974	0.1947
40	Urea	0.5273	0.7819	-0.1621
41	Uric acid	0.4404	0.7571	-0.1996
42	Valine	0.4063	0.7571	0.3406
43	Xylose	0.2911	0.6974	-0.1576

Table S8. Summary of statistical testing results for GC-MS urinary metabolites between the HC and IBS groups. The table presents raw *p*-values (Welch's *t*-test or Mann–Whitney U test), FDR-adjusted *q*-values, and effect sizes (Hedges' *g*)

No.	Metabolite	*p*-value	*q*-value	Hedges' *g*
1	1,5-Anhydroglucitol	0.3659	0.7993	0.0241
2	2-Aminoethanol	0.9590	0.9590	-0.1608
3	2-Deoxytetronic acid	0.5253	0.7993	0.3200
4	2-Hydroxybutyrate	0.3757	0.7993	-0.2930
5	3-Hydroxyisovaleric acid	0.3639	0.7993	-0.1725
6	3-Hydroxyphenylacetic acid	0.4671	0.7993	0.1260
7	6-Deoxyglucitol	0.4829	0.7993	0.1089
8	Alanine	0.2681	0.7993	-0.4196
9	Arabinose	0.5422	0.7993	0.2782
10	Ascorbic acid	0.9328	0.9590	0.2857
11	Citric acid	0.2244	0.7993	-0.2562
12	Creatinine	0.6088	0.7993	0.1311
13	Dehydroascorbic acid	0.3410	0.7993	0.2808
14	Dopamine	0.5157	0.7993	-0.2593
15	Fructose	0.5593	0.7993	-0.2188
16	Fumaric acid	0.6255	0.7993	-0.1428
17	Galactose	0.1810	0.7993	-0.1091
18	Glucose	0.0585	0.7993	-0.3917
19	Glyceric acid	0.8001	0.9041	0.2275
20	Glycerol-3-galactoside	0.2286	0.7993	0.2348
21	Glycine	0.9474	0.9590	0.1007
22	Glycolic acid	0.5644	0.7993	-0.0188
23	Hexose	0.4339	0.7993	-0.3494
24	Histidine	0.7552	0.8907	-0.0818
25	Isoleucine	0.5253	0.7993	-0.3388
26	Isothreonic acid	0.5611	0.7993	0.1490
27	Lactic acid	0.3354	0.7993	-0.2016
28	Lactose	0.2092	0.7993	-0.4330
29	Levoglucosan	0.5743	0.7993	0.4402
30	Lysine	0.9094	0.9590	0.0071

No.	Metabolite	*p*-value	*q*-value	Hedges' *g*
31	Lyxose	0.6946	0.8636	-0.1121
32	Mannose	0.0606	0.7993	-0.3517
33	Meso-Erythritol	0.2478	0.7993	0.2085
34	N-Methylethanolamine	0.2928	0.7993	0.2108
35	Oxalic acid	0.4739	0.7993	0.0777
36	Palmitic acid	0.8689	0.9517	0.0876
37	Pantothenic acid	0.5894	0.7993	-0.0472
38	Phosphate	0.0676	0.7993	0.5543
39	Propyleneglycol	0.5422	0.7993	0.2120
40	Psicose	0.5996	0.7993	-0.3082
41	Ribonic acid	0.1859	0.7993	0.2740
42	Ribose	0.5895	0.7993	-0.0323
43	Serine	0.8058	0.9041	-0.3451
44	Sorbitol	0.7302	0.8839	0.1928
45	Succinic acid	0.4492	0.7993	-0.0381
46	Threonine	0.1859	0.7993	-0.4235
